# Seaweed Phenolics as Natural Antioxidants, Aquafeed Additives, Veterinary Treatments and Cross-Linkers for Microencapsulation

**DOI:** 10.3390/md20070445

**Published:** 2022-07-07

**Authors:** Tharuka Gunathilake, Taiwo O. Akanbi, Hafiz A. R. Suleria, Tim D. Nalder, David S. Francis, Colin J. Barrow

**Affiliations:** 1 School of Life and Environmental Sciences, Deakin University, Geelong, VIC 3216, Australia; kgunathilake@deakin.edu.au (T.G.); tim.nalder@deakin.edu.au (T.D.N.); d.francis@deakin.edu.au (D.S.F.); 2 School of Environmental and Life Sciences, College of Engineering, Science and Environment, University of Newcastle, Ourimbah, NSW 2308, Australia; taiwo.akanbi@newcastle.edu.au; 3 Department of Agriculture and Food Systems, The University of Melbourne, Melbourne, VIC 3052, Australia; hafiz.suleria@unimelb.edu.au

**Keywords:** aquafeed, phenolics, seaweed, veterinary drug, antioxidant, cross-linker, extraction and characterisation

## Abstract

Driven by consumer demand and government policies, synthetic additives in aquafeed require substitution with sustainable and natural alternatives. Seaweeds have been shown to be a sustainable marine source of novel bioactive phenolic compounds that can be used in food, animal and aqua feeds, or microencapsulation applications. For example, phlorotannins are a structurally unique polymeric phenolic group exclusively found in brown seaweed that act through multiple antioxidant mechanisms. Seaweed phenolics show high affinities for binding proteins via covalent and non-covalent bonds and can have specific bioactivities due to their structures and associated physicochemical properties. Their ability to act as protein cross-linkers means they can be used to enhance the rheological and mechanical properties of food-grade delivery systems, such as microencapsulation, which is a new area of investigation illustrating the versatility of seaweed phenolics. Here we review how seaweed phenolics can be used in a range of applications, with reference to their bioactivity and structural properties.

## 1. Introduction

As the global population is projected to increase from almost 8 billion in 2022 to 9.7 billion in 2050, the demand for animal protein continues to rise [1,2]. Worldwide the percentage of fish stocks fished within biologically sustainable levels reduced from 90% to 65.8% from 1975 to 2015 [3]. Hence, aquaculture has grown rapidly to meet production needs as wild capture fisheries decline to ensure food security and protect marine biodiversity. Aquaculture now produces 50% of the world’s fish production intended for food [3]. Fisheries and aquaculture products are among the healthiest food sources on the planet, containing high levels of protein, omega-3 fatty acids and vitamin D. By 2016, the average rate of global fish consumption (3.2%) outpaced all terrestrial animal proteins combined (2.8%) [3]. In 2015, fish represented 17% of the animal protein consumed globally, fulfilling almost 20% of the average per capita animal protein intake for 3.2 billion people [3].

Despite the fast growth rate, the stability of aquaculture production shows a boom-and-bust pattern according to the FAO’s global database for farmed aquatic organisms from 1950 to 2015 [4]. Market problems and ecological limits are frequently discussed issues and these, plus diseases and phenomena, such as inbreeding depression, are major reasons for the rise and fall of seafood production [5]. Numerous studies have endeavoured to develop novel feed additives for safe and effective stress and disease management formulas [6].

Disease outbreaks significantly impact aquaculture production and lead to substantial economic loss [7], with exposure to sudden environmental changes such as water quality, temperature and inadequate nutrition exacerbating the situation [7]. The greatest concern regarding intensive fish farming is oxidative stress [8]. The production of excessive reactive oxygen species (ROS), such as superoxide (O_2_^−^) and peroxide (H_2_O_2_), result in macromolecular damage (protein, DNA, lipid and other biomolecules) and associated pathological symptoms that lead to negative growth performance and overall fish health [9]. To protect against oxidative damage, fish possess antioxidant systems that utilise enzymes, vitamins and minerals [10]. Fish also need to acquire certain antioxidant compounds (e.g., selenium) through their diet and environment, which cannot be synthesized in vivo [11]. Antioxidants in feedstuff aid in the conservation of fat-soluble vitamins, such as vitamin A and E, throughout digestion and absorption, which protect the animal from nutrient deficiencies [12]. Aside from animal health, antioxidants also prevent rancidity and lipid oxidation in feed formulations [8]. As previously reported, synthetic antioxidants (e.g., ethoxyquin/EQ) have been widely used in aquafeed formulas for many years. However, with mounting evidence of adverse effects on aquatic life and humans, the EU commission suspended the authorisation of ethoxyquin as a feed additive for all animal species and categories [13,14,15]. Accordingly, safe use requirements need to be followed for synthetic additives (<150 ppm for ethoxyquin) and a requirement for labelling on all products [16].

**Figure 1:**
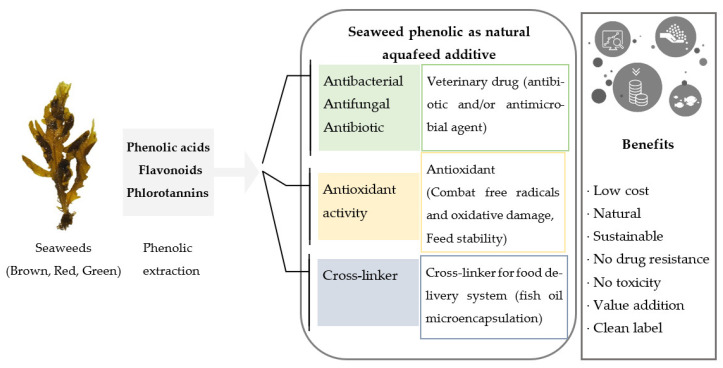
Schematic representation of seaweed phenolic compounds and their potential application as natural aquafeed additive with the intended benefits.

As prophylactic and therapeutic interventions, several veterinary drugs and antimicrobials have been used in aquaculture as additives in fish feed, injections and baths, which are strictly regulated [7]. Consequently, antibiotic resistance for fish pathogens has developed among aqua species and has also affected land animals and humans [17]. Diet formulation is a vital aspect of intensive farming and represents 50–70% of the total production cost, 20% of which can be attributed to synthetic additives [18,19]. Accordingly, the development of cost-effective and bio-safe natural feed additives from sustainable and renewable sources is of great interest in the aquaculture industry (Figure 1).

Over the past few decades, seaweed phenolics have become an attractive, sustainable source of antioxidants with an array of bio-functional properties, which could potentially substitute for existing aquafeed additives. The biological properties of a range of phenolic compounds from different seaweeds have been widely investigated, yet few attempts have been made to utilise these compounds in aquafeed formulations as bio-functional ingredients. Therefore, this review aims to provide an overview of the sustainability of seaweed cultivation, the phenolic profile of brown, red, and green seaweeds alongside their structural classification, an overview of multi-step systematic approaches for phenolic extraction, separation, and identification, with methodological comparisons relevant to industrial use. We also discuss the existing literature on seaweed phenolics, which are extracted from cultivated and wild harvest species from a range of locations worldwide, and their capacity to be used as natural veterinary drugs, antioxidants, and cross-linkers in aquafeed with the view of developing sustainable, high-quality and more stable feeds for aquaculture.

## 2. Seaweed Phenolics as Sustainable Aquafeed Additives

Acceptability and commercialization of novel aquafeed ingredients depend on the nutritional profile, cultivation cost, seasonality, and economies of scale [20,21]. Seaweed, also referred to as macroalgae, have been studied as potential aquafeed ingredients since the late 1970s [22] but have been implemented as a poultry feed supplement since the 1950s [23]. Some species of seaweed grow rapidly with no requirement for freshwater, arable land, fertiliser or pesticides, and are usually available year-round with some seasonal variations in growth [24]. The constant supply of biomass coupled with a high nutritional profile makes seaweed a sustainable choice for aquaculture and food security [25]. Seaweeds, along with microalgae, form the base of the aquatic food web, where nutrients pass through the food chain toward top-end predators such as sharks, dolphins, and whales [26]. As such, seaweeds contribute substantially to aquatic life, either directly or indirectly, by providing essential nutrients. However, to date, seaweed remain underutilised in aquaculture.

Previous studies suggest that most seaweeds can be fed to an aquatic animal as an additive or supplement [20,21], providing their dietary inclusion levels are not too high. The inclusion of seaweed in aquafeed has been attempted as either seaweed meal or seaweed extract [20]. This has been shown to provide general physiological improvements in animals, such as growth performance [27], feed utilisation [28], disease resistance, stress response [28], fillet quality, natural pigmentation, protein retention during winter, and increased long-chain polyunsaturated fatty acid concentration in fillets [28,29]. Therefore, preparing aquafeed formulations containing targeted seaweed-derived ingredients could promote fish health at a low cost. It has been reported that when the inclusion of seaweed in aquafeed is beyond 10% *w/w* it impacts negatively on most species [30]. Mechanistically it has been suggested that non-starch polysaccharides (cellulose and hemicellulose) and anti-nutritional factors (tannins, phytic acid, lectins, amylase, and trypsin inhibitors) present in seaweeds can reduce nutrient accessibility [31]. Extraction of bioactive compounds from seaweeds for inclusion into aquafeeds would help overcome the problems caused by the inclusion of the whole biomass, of which carbohydrates are generally the major component.

In addition to basic nutrients, seaweeds are relatively unexplored sources of numerous bioactive compounds (polyphenols, pigments, essential fatty acids, vitamins and amino acids). These are categorised as secondary metabolites, which are synthesised for a range of reasons, such as protective mechanisms against infections and environmental stress conditions [32]. With advances in separation science, a vast range of these compounds can now be isolated and characterised [33]. Recently, interest has grown considerably in using seaweed as a source of functional ingredients rather than whole macronutrient sources. Seaweed phenolics provide alternative ingredients that are complementary to synthetic additives used in aquaculture, possessing a broad spectrum of bioactive properties such as antimicrobial, antiviral, antifungal, anti-stress, antioxidant, anti-inflammatory, immunostimulant, and appetite stimulation [34]. Also, their antioxidant properties retard lipid oxidation and preserve feed quality improving shelf life. In addition, low molecular weight (LMW) phenolic compounds such as bromophenols (BP) (2-BP, 4-BP, 2,4-BP, 2,6-BP, 2,4,6-BP) are natural flavour compounds that can enhance fish fillet flavour and increase the market value [35]. According to Ma et al. [35], silver seabream-fed diets supplemented with seaweed showed significant deposition of bromophenol in fish flesh and gut, which imparted a “sea-like flavour” in the fillets.

This demonstrates that seaweed phenolics can be safe, sustainable and natural additives in aquatic health management, as well as having the added benefit of consumer appeal regarding the demand for natural products in food labelling. Further research is required to ensure that the potential of seaweed and the compounds they contain are realised.

## 3. Overview of Seaweeds

**Figure 2:**
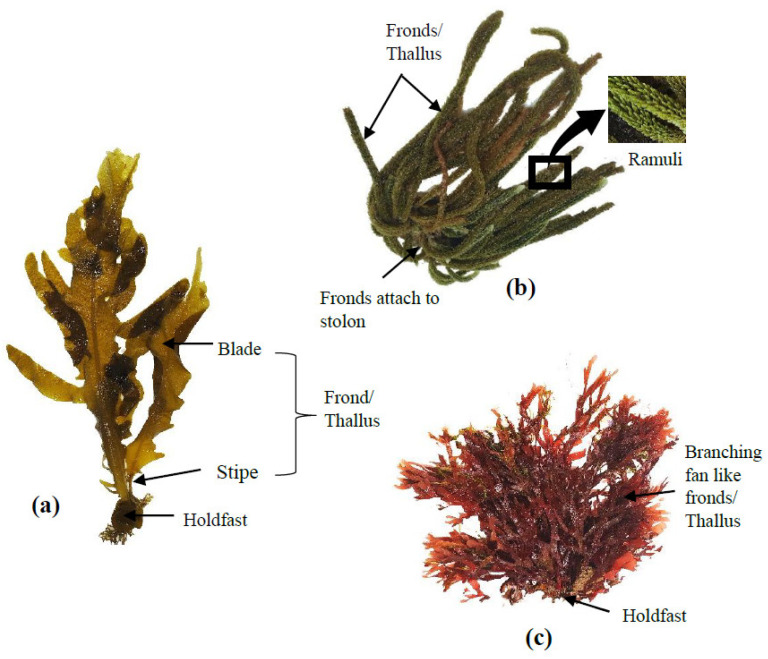
Seaweed thallus structures for (**a**) brown seaweed (e.g., Lessoniaceae), (**b**) green seaweed (e.g., Caulerpaceae) and (**c**) red seaweed (e.g., Galaxauraceae).

For centuries, seaweeds have been widely used in Asia (particularly in China, Japan, and Korea) as a traditional food source. The majority of global seaweed production (more than 80%) is consumed by humans as fresh or dried whole seaweed or used to produce food hydrocolloids (mainly agar, alginate and carrageenan). The remaining less than 20% are used for a range of industrial applications, such as feed ingredients (animal and fish feed), cosmetics, bioplastics and fertilisers [36]. Seaweeds fall into three taxonomic groups based on their thallus pigmentation: brown seaweed (Ochrophyta), red seaweed (Rhodophyta) and green seaweed (Chlorophyta) [37]. Brown seaweeds are long, thick, and leather-like species and can reach up to 45 m long [38]. Red seaweeds are relatively small (up to ~1 m) species with different shades, including red, purple, or brownish-red [37]. Green seaweeds are closely related to red seaweeds and are similar in size [37]. The basic thallus structures of brown, green and red seaweeds are shown in Figure 2. Seaweeds are typically found in estuarine intertidal and subtidal habitats and coastal areas, with some kelps being among the fastest-growing plants on earth [21].

Approximately 25,000–30,000 species of seaweed are known to exist worldwide [39]. Global seaweed production is currently worth over USD $6 billion per annum, with 85% of production for human consumption [40]. There are about 50 countries involved in commercial seaweed farming, with the most production in Asia (China, Indonesia, Philippines, Republic of Korea, Malaysia and Japan), South America (Chile), Europe (Denmark) and Africa (Tanzania) [40,41].

Seaweeds are also an attractive resource for integrated multi-trophic aquaculture systems (IMTA) as a food source for aquaculture species (e.g., sea urchins) that can provide a buffer against ocean movement and actively bioremediate organic waste produced from intensive fish farming practices [42]. IMTA is a viable approach in both sea-based aquaculture (salmon, bluefin tuna) and land-based farms (prawns, abalone, and finfish aquaculture) [42]. IMTA shows numerous benefits over monoculture systems, including multiple harvests, lowered production costs, large-scale viability and minimisation of environmental impact [21].

## 4. Seaweed Phenolics Compounds

### 4.1. Classification of Phenolic Compounds

**Figure 3:**
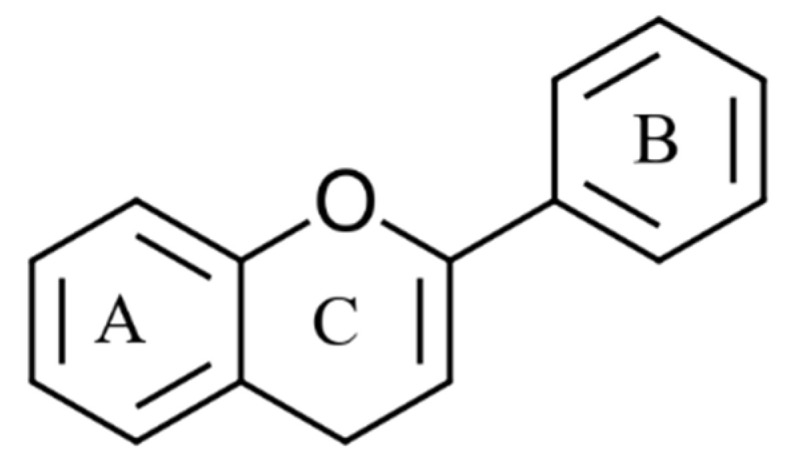
The basic skeleton of flavonoid compounds. A & B; phenyl rings and C; heterocyclic pyran ring.

Phenolic compounds are a highly heterogeneous group of compounds found in terrestrial plants and marine seaweeds [43]. These bioactive molecules contain at least one aromatic ring with a single hydroxyl group (–OH) [44]. So far, over 8000 phenolic compounds have been identified with structural diversity ranging from low molecular weight single aromatic ring monomers to highly complex polymerised structures [44]. In the literature, phenolics are divided into different categories based on their origin, structure, and functionality. To simplify, this review categorises phenolic compounds into two groups based on their chemical structures: flavonoids and non-flavonoids. Flavonoids are the most widely distributed group and account for nearly two-thirds of all known phenolics [45]. These molecules are made up of two phenyl rings attached to a heterocyclic pyran ring (Figure 3) [45]. By changing the structural properties of the pyran ring, flavonoids are further divided into six groups: flavones, isoflavones, flavanols, flavanones, flavonols (flavan-3-ol), anthocyanidins [45,46]. However, individual compounds in each of these groups vary by the methylation and hydroxylation patterns of the two phenyl rings [45].

**Figure 4:**
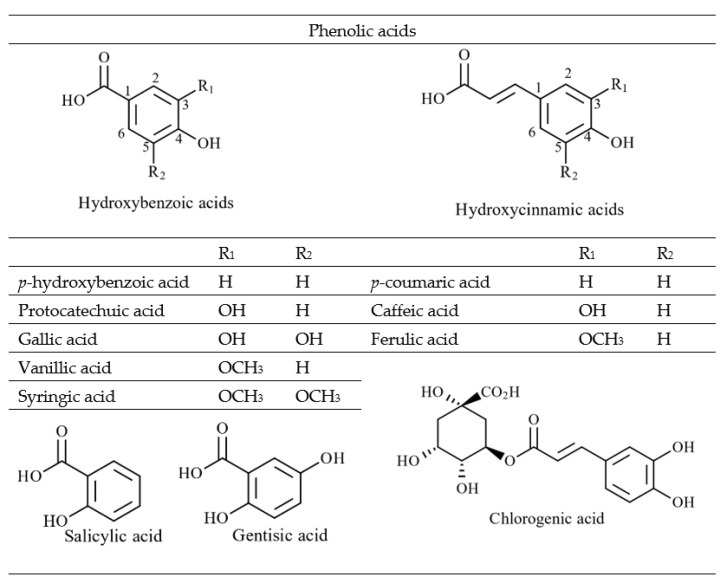
The basic skeleton of hydroxybenzoic acids and hydroxycinnamic acids found in seaweed.

Non-flavonoids are phenolic compounds consisting of a single phenyl group through to high molecular weight polymerised complexes [45]. Most are found in fruits and vegetables and are referred to as phenolic acids, commonly containing a phenyl ring bound to one or more functional groups [45]. Based on their derivatives, either benzoic or cinnamic acid, most phenolic acids are further divided as hydroxybenzoic or hydroxycinnamic acids, respectively (Figure 4) [46]. Additionally, other phenolic acids exist as hydroxyphenyl acids (acetic, propanoic and pentaenoic) [47]. Lignans, stilbenes and tannins are different groups of non-flavonoid compounds found in the plant kingdom [46]. The major flavonoids and non-flavonoid compounds found in seaweeds are discussed below.

### 4.2. Phenolic Compounds Found in Seaweeds

#### 4.2.1. Phenolic Acids and Flavonoids

**Figure 5:**
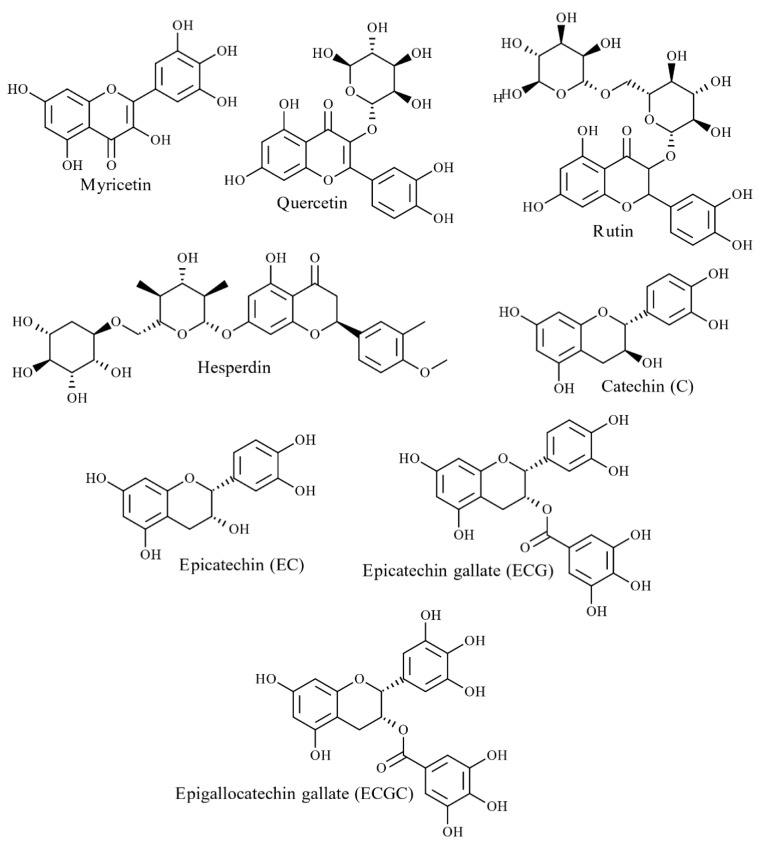
Structures of major flavonoids identified in seaweeds.

To date, numerous polymeric structures have been identified in red, green and brown seaweed species [33]. As reported previously, the major phenolic constituent of red and green seaweeds are flavonoids (Figure 5) and phenolic acids [33]. However, bromophenols (halogenated phenolics) mainly exist in red seaweeds, while phlorotannins are found exclusively in brown seaweeds [46]. Some studies report that phlorotannins are the only phenolic compound found in brown seaweeds [48], whilst others report the presence of flavonoids and phenolic acids [49,50,51,52]. Quantification analysis of *S. scoparium* (brown seaweeds) aqueous extract reported the presence of significant concentrations of phenolic acids and flavonoids; 90 mg/100 g dry weight (DW) of gallic acid followed by catechin and epicatechin (6–7 mg/100 g DW) [50]. Yoshie-Stark et al. studied flavonoid distribution in methanolic extracts of 27 Japanese seaweed species (6 green, 11 brown and 10 red seaweeds), revealing a high abundance of flavonoids in red seaweeds compared to green and brown seaweeds [52]. Hesperidin was found in all red seaweeds (626–119,000 µg/g) and some green and brown seaweeds [52]. Catechol was common in all green and red seaweeds (1660–77,700 µg/g) as well as most brown seaweeds [52]. Rutin and caffeic acids were distributed amongst all three groups but were most prominent in red seaweeds (23,200–4,000 µg/g) [52]. Quercitrin and myricetin were present in low concentrations in brown and red seaweeds (202–466 µg/g), whereas morin was detected in all seaweed samples in small quantities (257–3730 µg/g) [52].

Furthermore, twelve phenolic acids were reported in red and brown seaweed extracts: *Porphyra tenera* (nori) and *Undaria pinnatifida* (wakame), namely hydroxybenzoic acid (salicylic acid, 2,3-dihydroxybenzoic, *p*-hydroxybenzoic, protocatechuic), hydroxycinnamic acid (*p*-coumaric, caffeic, chlorogenic) and hydroxybenzaldehydes (3,4-dihydroxybenzaldehyde, *p*-hydroxybenzaldehyde) [53]. Further, Klejdus et al. [54] identified eight isoflavones (daidzin, daidzein, genistein, formononetin, sissotrin, biochanin A and ononin) in seven red and brown seaweeds, with the highest concentrations present in *Chondrus crispus* (red seaweed 86–229 ng/g) followed by *Halopytis incurvus* (red seaweed 7–50 ng/g) and *Sargassum muticum* (brown seaweed 7–144 ng/g) suggesting that these isoflavone compounds are restricted to specific seaweed species [33]. In addition to halogenated phenolics, sulphate metabolites have occasionally been observed in red, green and brown seaweed [33,55]. For instance, sulphated coumaric acids, benzoic acids and phenylacetic acid have been reported in the green seaweed *Dasycladus vermicularis* [56].

#### 4.2.2. Phlorotannin

Phlorotannin, like tannin in terrestrial plants, is a heterogeneous group of polymeric compounds found only in brown seaweeds [32]. They are biosynthesised through dehydro-oligomerisation and dehydro-polymerisation of phloroglucinol (1,3,5-trihydroxybenzene) units (PGU) via aryl-aryl (C-C) bonds and/or diaryl-ether (C-O) bonds, which produce molecules with masses ranging from 126–650 kDa [43,51]. Based on the position of polymerisation, different isomers exist [57] that create difficulty in the structural elucidation of seaweed extracts. Phlorotannins with 12 PGU in *F. vesiculosus* lead to 61 isomers demonstrating the complex nature of these compounds [58]. Unlike other phenolic compounds, this group is believed to have over 150 molecular structures due to different molecular sizes and linkages [59].

The presence of many phenolic hydroxyl groups makes them more hydrophilic. Furthermore, it contributes to the different roles they play in seaweeds, such as chelate divalent metals, integral structural constituents that bind with polysaccharides, protein and other biopolymers [57]. Concentrations of phlorotannins upwards of 15% in dry weight (DW) have been reported in brown seaweeds [60], with 12% DW reported in Fucus sp. and 14% DW in *A. nodosum* [32].

**Figure 6:**
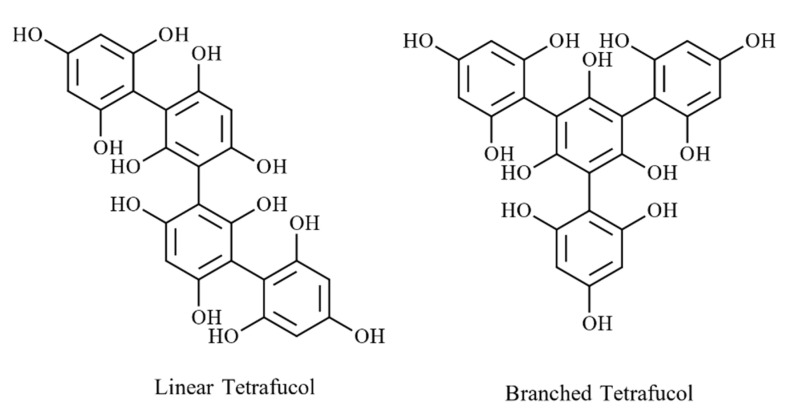
Examples for linear and branched phlorotannin (e.g., Tetrafucol).

**Figure 7:**
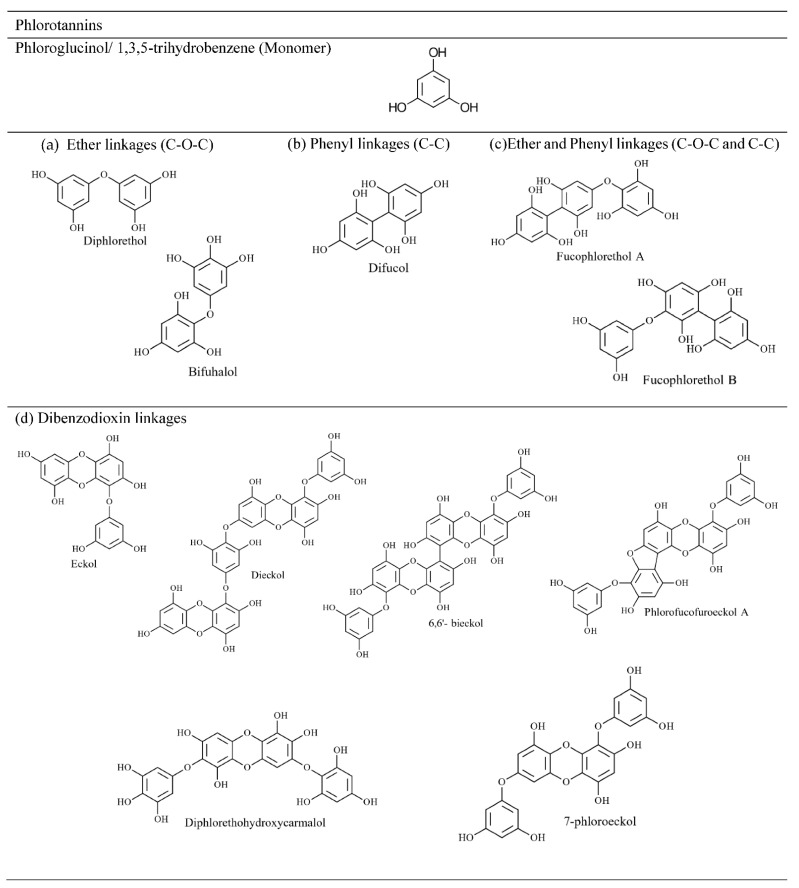
Phlorotannins classification based on type of linkage between aromatic units, (**a**) phlorethol and fuhalol, (**b**) fucol, (**c**) fucophlorethol, (**d**) eckol and carmalol.

The type of linkages between monomers can be used to classify phlorotannins into four groups: phlorethols and fuhalols with an ether linkage, fucols with a phenyl linkage, fucophlorethols with ether and a phenyl linkage, and eckols and carmalols with a benzodioxin linkage [61]. Compounds in all four groups can be further divided into linear (bound only to two phloroglucinol units) and branched phlorotannins (bound to three/more phloroglucinol units) [57]. Figure 6 depicts the linear and branched arrangements of tetrafucol in *Fucus vesiculosus* [62], while Figure 7 shows major classes of phlortannins.

Significant amounts of phlorotannins have been recorded in Laminariales (*Ecklonia* spp. and *Eisenia* spp.), Fucaceae (*A. nodosum* and *F. vesiculosus*) and Sargassaceae families [51,63]. Previously, phlorotannins found in brown seaweeds have been reported by compound type rather than subclass [43], with some authors claiming that fucols and fucophlorethols are prominent among the Fucaceae family [63]. Accordingly, phloroglucinol, eckol (including carmalol derivatives), dieckol, 6,6′-bieckol, fuhalol, 7-phloroeckol and phlorofucofuroeckol, and fucophloroethol, have been frequently identified in brown seaweed species [64,65]. Using conventional molecular characterisation techniques, the size of phlorotannin compounds was reported to be between 2 and 8 PGU [66]. However, using rapid phenolic profiling chromatographic and mass spectrometric techniques, larger phlorotannin compounds of 16 PGU [58] and 17 and 27 PGU [67] repeating units have been identified. Steevensz et al. [68] successfully investigated rapid phlorotannins profiling methods using ultra-high-performance liquid chromatography-high-resolution mass spectrometry (UHPLC-HRMS), enabling the characterisation of phlorotannins with 3–49 PGU in *P. canaliculata*, which is by far the highest level of polymerisation recorded in brown seaweed. However, low molecular weight phlorotannins preferentially have 4–12 PGU according to Ultra Performance Liquid Chromatography–Triple-Quadrupole Mass Spectrometry (UPLC-QQQ-MS) data in Fucaceae and Himanthaliaceae species [58].

### 4.3. Occurrence and Biosynthesis

Phenolic compounds in seaweeds originate as secondary metabolites and are available in two forms [69]. Soluble forms protect against stress conditions, herbivory, heavy metal exposure and oxidative damage emanating from UV radiation and nutrient deficiency [70]. Insoluble forms, also known as cell wall-bound polyphenols (mostly phlorotannins), usually bind with alginic acid and protein via ester covalent bonds [71]. Phlorotannin with structural polysaccharides plays a vital role in the structural development of brown seaweed cell walls [72]. However, quantification studies of brown seaweed phlorotannin from *F. vesiculosus* show that their soluble form in the cytoplasm was more abundant than the cell wall-bound form [73].

Phenolics accumulate in membrane-bound vesicles called physodes in the cytoplasm of seaweed cells [70]. Physodes are produced in the endoplasmic reticulum and Golgi apparatus before being transferred to fuse with the cell membrane and begin phenolic secretion [70]. Baardseth, E. [74] reported that physodes represent 3–11% *v/v* of the seaweed body of *F. vesiculosus*, *A. nodosum* and *F. serratus*. Based on reports from *Laminaria hyperborea* and *Fucus serratus* species, the size of physodes is relatively uniform at ~2,500 μm [75]. The study between three brown seaweed species revealed phlorotannin is exclusively produced in the peripheric vegetative cells of thalli [76]. Phlorotannins are synthesised due to polymerisation of phloroglucinol (1,3,5-trihydroxybenzene) monomers [59]. The biosynthesis of phloroglucinol happens via the acetate-malonate (polyketide) pathway utilising the type III polyketide synthase enzyme followed by oxidative polymerisation process to form phlorotannins [77].

### 4.4. Variation of Phenolic Content

The phenolic content of seaweeds varies and is dependent on many factors. The bioactive properties of these compounds help seaweeds survive under the harsh conditions of the marine environment [43]. Phenolic compositions vary interspecies and/or intraspecies, and sometimes even within a single species thallus [43]. Intraspecies phenolic composition varies according to specific intrinsic (thalli size, reproductive state, age, tissue type) and extrinsic factors (temperature, light, contaminants, nutrients availability, salinity and geography) [78]. Additionally, harvest season, location and extraction methods are known to affect the phenolic profile. Due to the high variability of these factors, it becomes difficult to compare interspecies and/or intraspecies differences in phenolic composition.

Compared to red and green seaweeds, brown seaweeds possess more phenolic compounds, particularly phlorotannins [79]. The phenolic content in brown seaweed is influenced by species, location, and season. Connan et al. [80] studied the variation among eight brown seaweed species across 14 months, revealing greater phenolic content in *Fucales* sp. (>2% DW) in comparison to *Laminaria digitata* as a result of genetic adaptation to environmental factors. They also found that species occupying the middle intertidal zone exhibited the highest phenol concentration (~5.8% DW) compared to lower and upper levels of the shore (0.03–1.40%) [80]. Seaweeds in different intertidal zones are subject to different environmental conditions such as light intensity, salinity, temperature, and periods of dehydration [80]. Regarding seasonal variation, there was a taxonomic difference among the species studied. The highest phenolic content was observed in Fucaleans in summer, given the need for photo-protective mechanisms, whereas Laminariales demonstrated the maximum amount of these compounds in winter [80]. Similar results demonstrated the highest level of phlorotannin excretion in *Ascophyllum nodosum* (member of Fucaceae family) in summer [81]. The correlation between phenolic excretion and light intensity was also observed by Abdala-Díaz et al. [82]. Accordingly, the phenolic concentration of brown seaweed (*Cystoseira tamariscifolia*) was the highest in the apical section of the thallus. It decreased towards the middle and basal sections, given they were tightly packed against each other and shaded from UV and photosynthetically active radiation [82]. The variation of phlorotannin content with respect to reproductive status had been studied for *Ecklonia cava*, having phlorotannin levels 1.5 times higher in mature thalli than in younger samples [57]. Salinity also appears to have a positive relationship with phenolic excretion [83]. For instance, studies of *F. vesiculosus* and *A. nodosum* showed an increase in phenolic content as the salinity of surrounding water increased [84].

The extraction method employed for analysis is also a factor that significantly impacts the total phenolic content (TPC) of seaweed samples. According to Dang et al. [85], novel phenolic extraction methods, such as ultrasonication-assisted extraction, gave higher yields than conventional extraction methods. However, the vast range of inter- and intra-species phenolic variation is problematic for the use of these products in aquafeed formulations, as difficulties arise in obtaining standard phenolic composition in the final products, particularly when harvested from natural habitats [86]. On the other hand, this presents an opportunity for seaweed farmers to change and maximise phenolic composition based on their intended requirements through stimulating growth conditions and selective breeding programs [21,87].

## 5. Extraction and Identification of Seaweed Phenolic Compounds

Extraction and identification of phenolic compounds from seaweed samples is a difficult task given the complex nature of the seaweed matrix. High abundances of fucose-containing sulphated polysaccharides and alginates embedded in algal cell walls make cross-links to cellulose [88]. Moreover, phlorotannin forms covalent and non-covalent bonds with proteins [89]. These structural components act as a physical obstacle to the release of phenolic compounds during extraction [90]. In addition to interfering compounds, the extract yield of seaweed phenolics depends on several factors, including the extraction method, solvent type and ratio, extraction time, temperature, the physical state of the seaweed sample (raw sample, dried powder, particle size, etc.) and storage conditions (light, temperature, humidity, oxygen, all of which can cause oxidative deterioration of phenolics) [91]. Thereby, sequential extraction, separation, purification and characterisation steps have been proposed for effective extraction and identification of phenolics [33,75].

### 5.1. Sample Handling and Pre-Treatments

Before seaweeds, and the compounds they contain can be analysed and characterised, their biomass must be appropriately handled and pre-treated. Firstly, collected seaweed samples from coastal areas are rinsed with fresh or deionised water to remove residual salt, sediments, sands, invertebrates, and epiphytes [92]. Secondly, residual water is removed by draining, and samples are then subjected to different drying techniques, such as sun drying, conventional oven drying (from 40 to 60 °C) and freeze=drying (from −50 to −80 °C). This is done to prevent possible ongoing biochemical reactions and makes packaging and storage for extraction and characterisation purposes much less laborious [69,92,93,94]. The optimum oven drying temperature had been suggested as 40 °C for maximum TPC and TAA (total antioxidant activity) concentrations, a balance must be found as low temperatures release moisture more slowly, whereas higher temperatures can degrade bioactivity [69]. Finally, samples are ground or milled to increase surface area [95]. The method of drying has a significant effect on the TPC and the antioxidant properties of seaweed samples. Previously, it has been shown that oven drying of *Sargassum* sp. was more effective than freeze- and sun-drying [96]. Conversely, Cruces et al. [97] reported higher TPhC (total phlorotannin content) yield from freeze-dried *Lessonia spicata* (kelp) extract compared to other drying methods (sun-dried, oven-dried, silica-dried).

### 5.2. Extraction of Seaweed Phenolics

Extraction of phenolic compounds from seaweeds can be achieved using a range of approaches and technologies. For the purposes of this review, we have divided them into ‘conventional solvent extraction’ and ‘novel extraction’ methods.

Conventional solvent extraction: Traditional solid–liquid extraction (SLE) is the most common technique employed to extract phenolic compounds from seaweeds in a solvent media [33]. The efficacy of the SLE method is greatly determined by several parameters, including phenolic solubility in the solvent system (or polarity of solvent), solid–liquid ratio, extraction temperature, and time [33,75]. Among them, solvent selection is vitally important to obtain high yields of bioactive material. Notably, binary solvent systems, such as alcohol (ethanol, methanol) or acetone in aqueous media, are superior to individual solvents [64,65,98,99,100,101,102].

According to Farvin and Jacobsen [98], ethanol tends to be more efficient than water for phenol extraction. This report is consistent with Jiménez-Escrig et al. [99], as phenolic compounds of all species were found to be higher in polar organic extracts (methanol/water and acetone/water) when compared to aqueous extracts. Polar solvents (acetone) preferentially break down H bonds between phenolics and carboxylic (COOH) groups in proteins and facilitate greater leaching of phenolic compounds [103]. Accordingly, the extraction efficacy of phenolic compounds increases as solvent polarity increases [73]. Apart from being highly soluble, ethanol is recognised as a food-grade solvent and non-hazardous to human health, while acetone is not permitted in the food industry [65]. Regarding extraction, the highest yields have been reported for phenolic extracts using water compared to organic solvents [99]. A possible reason for this is that anionic sulphates, in the form of sulphated polysaccharides, are highly soluble in aqueous media giving rise to higher extraction yields [99,104].

Most importantly, several crude extracts and their fractions showed higher antioxidant activity despite low TPC content [86,98,102]. This suggests that co-extract bioactive, such as tocopherols, pigments, amino acids, and polysaccharides have antioxidant properties. In addition, nonpolar solvents (ethyl acetate, hexane, chloroform, diethyl ether) possess greater radical scavenging activity than polar solvents (butanol and methanol), reflecting their ability to extract terpenoids, fatty acids and flavonoids [105]. In addition to the choice of solvent, the extraction parameters most frequently investigated by previous studies are temperature (25 °C or room temperature), time (24 h) and solid–liquid ratio (1:10 or 1:20 *w/v*) [33]. The extraction parameters should be carefully selected to obtain maximum yields while conserving the integrity of phenolics; for instance, much higher extraction temperatures lead to the degradation of phenolic compounds [106].

Novel extraction methods: Although SLE is thought to be more convenient and feasible, novel and more sustainable approaches have already been proposed to eliminate the drawbacks associated with conventional phenolic extraction methods. Problems, including the requirement for large quantities of organic solvents, laborious processing, and the co-extraction of interfering molecules all lead to greater cost and social and environmental impacts [33]. Natural deep eutectic solvents (NADES) are a promising alternative to polar organic solvents. Recently, they were used to extract hydrophilic phenolic and phlorotannin compounds from *Fucus vesiculosus* and were found to be more effective than conventional SLE with ethanol [107].

The most popular alternative methods to conventional SLE are enzyme-assisted extraction (EAE), pressurised liquid extraction (PLE), microwave-assisted extraction (MAE), ultrasound-assisted extraction (UAE) and supercritical fluid extraction (SFE). A brief comparison of using these novel methods over SLE in phenolic extraction has been discussed below [33,75].

EAE is a green approach where several digestive enzymes (proteases and/or carbohydrases) are used to break down macromolecules in the seaweed matrix, allowing more phenolics to be solubilised [90]. An advantage of EAE is that it uses aqueous media to provide optimum conditions for enzyme activity [33].

PLE, also known as an accelerated solvent extraction (ASE) or subcritical water extraction (SWE), uses water under high temperature and pressure to increase the extraction efficacy of seaweed phenolics [75]. The water is pushed beyond its boiling point but remains in a liquid state via the elevated pressure in the system [75]. The higher temperature increases the solubility of the material being extracted from, thus facilitating diffusion and penetration of the solvent [108]. Surprisingly, these conditions have no impact on the oxidation of phenolic compounds due to the lack of other interferences such as light and air [106]. However, the high temperature makes Maillard and caramelisation reactions possible in seaweed samples [109].

MAE and UAE (non-thermal extraction) are also promising phenolic extraction methods that are considered pre-treatment techniques [75]. Here, seaweed samples submerged in solvent systems are subjected to microwave and ultrasound irradiations, respectively, thus disrupting biomolecules in the seaweed matrix and enhancing solvent penetration [33].

SFE is an excellent method for the extraction of heat-sensitive bioactive compounds [110]. Supercritical carbon dioxide (SC-CO_2_) is often used as the solvent in SFE as it is cheap, non-toxic, readily available, and environmentally friendly [111]. In comparison to organic solvents, supercritical fluids have a low viscosity, high diffusion rate and negligible surface tension, making them excellent for phenolic extraction from biomaterials [112].

### 5.3. Separation and Purification of Phenolics from Crude Seaweed Extracts

Crude seaweed extract contains phenolics and various compounds with diverse structures, including polysaccharides, pigments, proteins, steroids and fatty acids. Thus, detailed analysis and structural characterisations of phenolic constituents are difficult to achieve at this stage. In general, the Folin–Ciocalteu method, in line with in vitro antioxidant assays, is an inexpensive and convenient method used extensively for both qualitative and quantitative estimates in preliminary analysis and screening of phenolic content in crude seaweed extracts. However, non-phenolic reducing substances detected using the Folin–Ciocalteu method can overestimate TPC [75]. As such, quantitative nuclear magnetic resonance (qNMR) is required to provide relatively accurate data in complex mixtures and receives much more attention [75].

Seaweed extracts are normally subject to separation processes such as flash chromatography or high-performance liquid chromatography to separate and purify phenolic molecules or fractions before structure elucidation. Phenolic molecules can precipitate due to poor solubility and also may bind irreversibly during chromatography and so multiple chromatographic separations steps are normally required and yields can be low [33]. During purification, the crude extract is separated into different fractions based on solubility, molecular weight, charge or chemical affinity [33]. Liquid–liquid partitioning (LLP) has mostly been reported for the isolation of phlorotannin-rich fractions. As reported by Wang et al., ethanolic extract of *Fucus vesiculosus* was subjected to LLP with n-hexane to remove hydrophobic substances and a subsequent ethyl acetate extraction resulted in a phlorotannin-enriched ethyl acetate fraction [113]. It was further fractionated using column chromatography (solid-phase extraction (SPE)), and characterisation was performed by LC-MS, which tentatively detected phlorotannin oligomers [113,114].

### 5.4. Identification and Structural Elucidation

Tandem mass spectrometry (MS/MS) combined with high-performance liquid chromatography (LC/HPLC) and nuclear magnetic resonance (NMR) spectroscopy are platform solutions that enable simultaneous separation and identification of seaweed phenolics [64]. The phenolic fraction obtained through SPE needs to pass through the preparative chromatographic process (LC/HPLC/TLC), sometimes equipped with a UV detector or photodiode array (PDA), for effective detection and quantification of phenolic compounds [115]. Identifying phlorotannins is difficult due to the absence of standards, except for phloroglucinol monomers; thus, MS data is often utilised to compare theoretical mono-isotopic masses with that equivalent to phlorotannins [75]. Accordingly, chromatographic-mass spectrometric platforms tentatively indicate the structural diversity of phlorotannin components based on molecular size and degree of polymerisation [113].

Phlorotannin structures with the same molecular weight but with different PGU linkages are called isomers and require further verification via NMR analysis to elucidate their structures [116]. However, this requires pure samples in milligram quantities [117]. Proton (1H) and carbon (13C) NMR are the most common approaches, while two-dimensional NMR (hetero-nuclear multiple bond correlation; HMBC) can also be useful [118].

## 6. Seaweed Phenolics as Veterinary Treatments in Aquaculture

The use of seaweed phenolics in aquaculture as potentially therapeutic and biologically active compounds has received much attention. It is widely reported that their antibacterial, antifungal and antiparasitic activities protect against a range of pathogens responsible for stock and productivity loss (Table 1), as well as food borne pathogens (Table 2). In addition, marine seaweeds are rapidly becoming an appealing source of anti-inflammatory, neuroprotective, anti-hyperglycemic, anti-genotoxic and anti-carcinogenic phenolic compounds, which could provide alternative therapeutic agents in veterinary medicine applications (Table 3). However, there is currently limited understanding of how seaweed phenolics can supplement aquaculture feeds, with the goal of replacing synthetic additives, which often cause drug resistance and extensive feed cost.

According to previous studies, compounds extracted from brown seaweeds are more potent antimicrobial agents than those from red and green seaweeds [86,120,122,123,124]. For instance, phenolics from *Sargassum* spp., *Padina* spp. and *Ecklonia* spp. are highly active against Gram-negative fish and shrimp pathogens, such as *Vibrio* spp. and *Aeromonas* spp. Moreover, species such as *Ascophyllum* spp., *Fucus* spp., *Himanthalia* spp., *Padina* spp. and *Turbinaria* spp. Possess phenolics that are likely to be effective on foodborne bacteria associated with food poisoning [126,127,128,129]. In addition to antibacterial qualities, there is evidence supporting the antifungal properties of brown seaweeds; *Sargassum tenerrimum*, *Turbinaria ornate* and *Padina Pavonica* against commonly known pathogenic fungi (Table 2) [130,131]. This confirms that seaweed phenolics are not only natural antibiotics and antifungal supplements for aquatic species, but they can also act as marine-derived preservatives that prevent microbial spoilage of high-quality aquafeeds and prolong shelf life.

The general antimicrobial mode of action of phenolic compounds has been observed, which includes cytoplasmic membrane damage, NADH-cytochrome c reductase inhibition, ATP synthase inhibition and topoisomerase inhibition [144]. Seaweed phenolic, particularly phlorotannin, possess numerous hydroxyl groups (-OH in phloroglucinol units) which have a high affinity to bind with proteins (-NH groups) in bacterial cell membranes via hydrophobic interactions and hydrogen bonds, eventually triggering cell lysis [145]. Consequently, intracellular molecules leak out and disrupt membrane functions, including nutrient acquisition, oxidative phosphorylation and enzymatic functions (membrane proteins) [146]. It has also been reported that the presence of methyl- or acetyl-vinyl structures (tertiary structures) of phloroglucinol compounds possess high bacteriolytic activity [147]. Similarly, low molecular weight phlorotannins (LMPs) from *Sargassum thunbergii* appear to be effective against *Vibrio parahaemolyticus*, suggesting the possible use of LMPs as antimicrobial agents [122]. *Vibrio parahaemolyticus* is a halophilic Gram-negative bacterium that thrives in warm marine environments, which is problematic for aquaculture in these settings [148]. Most Gram-negative bacteria show antibiotic resistance as they possess specialised lipopolysaccharides (LPS) in their outer membranes, which act as a barrier to many antibiotics [149]. Therefore, phenolics with high bacteriolytic activity are required to combat Gram-negative species, such as *Vibrio* spp., compared to gram-positive bacteria.

It has been noted in previous investigations that determining whether the antibacterial activity of crude seaweed extracts is due to particular molecules or a range of molecules and their synergic effect [150]. In some cases, crude seaweed extracts have performed better than purified polyphenol fractions due to the presence of co-extracted molecules [86]. Thanigaivel et al. [125] found fish survival rate was significantly improved in an environment with the disease-causing fish pathogen *Aeromonas salmonicida*. This was due to a number of chemical compounds, properties and functional groups (aliphatic, cellulose, lignin, carboxylic, amides, alcohol, esters) present in the seaweed extract in addition to phenolic groups. GC-MS data of red and brown seaweed extracts showed a range of bioactive compounds (phenol, fatty acids, terpenes, volatile halogenated hydrocarbons, indoles and acetogenins) were responsible for the perforation of the cell wall of multidrug-resistant bacteria [151].

## 7. Seaweed Phenolics as Natural Antioxidants in Aquafeed

With rising demand, novel aquaculture management strategies are shifting toward therapeutic nutrition for sustainable productivity. Seaweed phenolics have mostly been reported as marine-origin antioxidants for aquafeed, promising ‘clean and green’ labelling on intended products. The antioxidant property of seaweed phenolics is by far the most promising bioactivity discussed this far. The Food and Agriculture Organisation (FAO) declared that antioxidants are a class of additives that prevent nutrient depletion and the rancidification of fats [12]. Uncontrolled oxidation leads to the destruction of biomolecules, such as pigments (carotenoids), fat-soluble vitamins (A, D, E) and amino acids, causing a reduction in feed consumption while also decreasing the nutritional values of the diet [12]. As a result, there is a need to study the relationships between active molecules and the antioxidant capacity of phenolics from various seaweed species, particularly in model systems. Establishing standard extraction and production techniques is also required for sustainable and cost-effective aquafeed formulation.

### 7.1. Importance of Antioxidants in Aquaculture Management

#### 7.1.1. Combatting Free Radicals and Oxidative Damage

Aquaculture systems are frequently exposed to stressors encountered via pathogens, chemotherapeutants and sudden environmental changes (e.g., temperature, salinity, UV radiation and heavy metal contamination) [8]. As a result, a set of chemically active molecules, including hydroxyl radicals (OH), superoxide radicals (·O_2_^-^) and other non-radicals such as singlet oxygen (^1^O_2_) and hydrogen peroxide (H_2_O_2_), can be formed, which are known broadly as reactive oxygen species (ROS) [152]. These ROS are by-products of the oxidative phosphorylation process (aerobic energy production), and the cell’s oxidative signalling network responds to them to regulate cellular metabolism and respond to environmental stressors [152]. In general, these reactive species are kept under control by endogenous cellular antioxidants, a natural defence system, to prevent the oxidative destruction of cell membranes and biomolecules, such as lipid, proteins and DNA [153]. However, in aquaculture, this equilibrium shift towards the production of excessive ROS levels due to a range of biotic and abiotic stress conditions known as “oxidative stress” [8]. Therefore, dietary supplementation with exogenous antioxidants is vital for maintaining the balance between ROS and antioxidants while minimising the adverse effects of oxidative stress [8].

#### 7.1.2. Feed Stability and Prolonged Shelf Life

**Figure 8:**
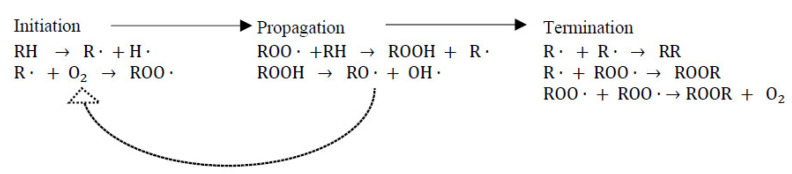
The general autooxidation process of polyunsaturated fatty acid.

Fish oil and fishmeal are important inclusions in commercial aquafeed. They are also readily oxidised given their high concentration of long-chain polyunsaturated fatty acids (PUFA), with rancidity resulting in unpleasant flavours, colours, odours and reduced nutritional value [154]. Lipid oxidation occurs via a series of molecular reactions, which are divided into three phases: initiation, propagation, and termination (Figure 8) [75]. Based on the mode of initiation, there are three types of lipid oxidation, including autoxidation, photo-oxidation and enzymatic oxidation [75].

Initiation: Autoxidation is more prevalent in lipids sources containing PUFAs and is usually initiated by exposure to either heat, light, trace metals, ionising radiation or existing free radicals [155]. In autoxidation, unsaturated lipid radicals (R·) form via the loss of hydrogen atoms from lipid alkyl chains (RH), which then further react with oxygen to produce peroxyl radicals (ROO·) [75]. Photo-oxidation is accelerated by exposure to light (radiant energy) and photosensitisers (pigments) of unsaturated fatty acids in the presence of oxygen [156]. Enzymatic oxidation involves lipoxygenase enzymes that incorporate molecular oxygen into PUFAs to form fatty acid hydroperoxides [31].

Propagation: During propagation, the formed peroxyl radicals (ROO) undergo chain reactions by uptake of hydrogen atoms from other polyunsaturated lipids and continuously produce lipid alkyl radicals (R·) and hydroperoxide (ROOH) [157].

Termination: Chain reactions are stopped by reacting intermediate free radicals with each other to produce non-radical compounds [75,155]. Lipid hydroperoxides formed during propagation are unstable and decompose into an array of secondary oxidation products, such as alcohols, hydrocarbons, aldehydes and ketones [155].

When these oxidation processes occur, aquafeeds become unacceptable for use, primarily due to the depletion of nutrients (mainly fat and protein) and oxidation products are potentially harmful to animal health [102]. Additionally, rancid feed can result in lower feed uptake by the species being farmed. Antioxidants are the most convenient solution to inhibit oxidative deterioration during processing and storage, reduce wastage, handling and storage costs while maintaining the nutritional value of the feed [155].

### 7.2. Synthetic Antioxidants in Aquafeed and Their Future Viability

**Figure 9:**
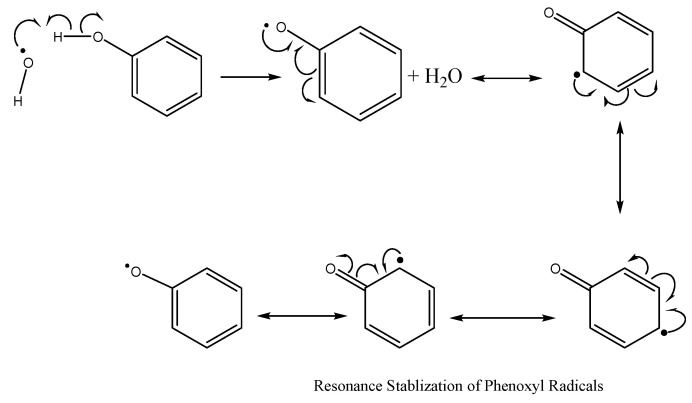
Formation of phenoxyl radical and resonance stabilization; the curved arrows represent the direction of the flow of single electrons.

There are two types of antioxidants, with these being defined by their mode of action [75]. Primary antioxidants, also called chain-breaking antioxidants, suppress free radical formation by transforming free radicals into more stable non-radical compounds. Alternatively, secondary antioxidants prevent lipid oxidation through various mechanisms, including decomposing hydroperoxides, oxygen scavenging, chelating transition metals (Fe^2+^, Cu^+^) and singlet-oxygen quenching in photo-oxidation [116]. Phenolic compounds are efficient primary antioxidants due to the presence of hydroxyl groups (OH). Notably, phenolics scavenge free radicals (e.g., hydroxyl and peroxyl radicals) via donating hydrogen atoms and becoming phenoxyl radicals, which are relatively stable due to resonance stabilisation when electron delocalisation occurs through the benzene ring (Figure 9) [116]. The mechanism of resonance of stabilization of phenoxy radical is shown in Figure 9.

Synthetic antioxidants such as butylated hydroxyanisole (BHA), butylated hydroxytoluene (BHT), tertiary butyl hydroxylquinone (TBHQ) and ethoxyquin (EQ) have been used extensively over the past three decades, with EQ being the most common [8,155]. However, the safety of EQ in target animals, humans and the environment has been questioned. Risks associated with residue, liver and kidney damage, carcinogenicity, and mutagenicity, are also potential issues associated with the use of this product [158,159,160].

The European Commission has placed a total ban on the use of EQ in feed manufacturing for all types of animal products [161]. Meanwhile, the Code of Federal Regulations (CFR) still permits EQ as a feed additive up to 150 ppm under specific safety rules and labelling requirements [162].

EQ had been widely used in feed preparation for decades. The sudden withdrawal of the ingredient had a negative impact on the aquaculture industry due to the lack of effective alternatives to EQ, the high cost associated with existing additives, and the failure to meet market specifications for certain animal products [13,163]. Several natural antioxidants, such as ascorbic acid, tocopherol and β-carotene can act against lipid oxidation, but they are costly and typically less effective in complex food/feed systems [116]. As such, there is a need for effective alternative antioxidants due to safety concerns and the global trend of natural ingredients.

### 7.3. Antioxidant Capacity of Seaweed Phenolic Compounds

Seaweed phenolics are a promising source of natural antioxidants for the feed industry, owing to their preserving effects at low concentrations [164]. An overview of the in vitro antioxidant properties of seaweed extracts/ fractions from an array of seaweed species is outlined in Table 4. TPC, along with in vitro antioxidant assays, are reported as the preferred methods for screening the antioxidant potential of seaweed extracts due to their convenience and cost-effectiveness [75]. TPC is often evaluated using a spectrophotometric Folin–Ciocalteau assay and results are expressed as either an equivalent quantity of gallic acid (GAE) or phloroglucinol (PGE) [165]. In vitro antioxidant assays are based on two modes of action: either a hydrogen atom transfer mechanism or a single electron transfer mechanism [155]. 

The total radical trapping antioxidant parameter (TRAP), oxygen radical absorbance capacity (ORAC) and crocin bleaching assay (CBA) are hydrogen atom transfer-based methods that donate H atoms to quench free radicals. In contrast, single electron transfer-based methods include 2,2′-diphenyl-1-picrylhydrazyl (DPPH), ferric reducing antioxidant power (FRAP) and trolox equivalent antioxidant capacity (TEAC) assays measuring antioxidant activity via transferring electrons to reduce metals, radicals, carbonyls, etc. [182]. In many cases, antioxidant assays positively correlate with the total phenolic content (TPC) [65,168,170]. However, co-extracted non-phenolic reducing substances can also react with Folin–Ciocalteu reagents and result in the overestimation of TPC values [75]. This can be overcome by using more specific quantification methods, for instance, quantitative nuclear magnetic resonance (qNMR) [183]. Moreover, seaweed extracts can be found with relatively low phenolic content and high antioxidant activity, explained by the presence of co-extracted compounds with antioxidant potencies, such as sulphated polysaccharides, and tocopherols, proteins or peptides and carotenoid pigments [49,98,99]. Therefore, comparing the antioxidant properties of seaweed extracts in line with TPC values is troublesome. To avoid such complications, this review only considers seaweed samples with high TPC and high antioxidant activities (Table 4).

Phlorotannins; Brown seaweeds contain molecules with relatively high levels of antioxidant activity, followed by red and green seaweed (Table 4). For example, brown seaweeds, including *Fucus, Bifurcaria, Ascophyllum, Ecklonia, Himanthalia, Sargassum, Macrocystis* spp., species have been reported to have high antioxidant potency (Table 4). The unique molecular structures identified in phlorotannins are the main contributor to most brown seaweeds’ antioxidant activity [184]. Phlorotannins are composed of up to eight interconnected benzene rings and are a good source of free radical scavengers compared to tannins derived from terrestrial plants that possess tannins containing only three to four interconnected phenyl groups [185]. In addition, phlorotannin also has metal chelating properties, reduction power, lipid peroxidation inhibitory activity, H_2_O_2_ scavenging activity and cellular antioxidant activity [49,64,69,99,167,169].

In general, samples with high TPhC are more potent antioxidants. As previously reported, *F. vesiculosus* found in the Arctic region are a rich source of phlorotannins and exhibited a strong positive correlation between TPhC and anti-radical power (Pearson’s correlation coefficients r = 0.64) [107]. Several studies have undertaken detailed characterisation and structural elucidation of phlorotannin composition using modified HPLC-MS/MS methods. For example, Li et al. found 42 types of phlorotannin compounds in an ethyl acetate fraction of *S. fusiforme*, which was found to have higher scavenging activity than Trolox (a synthetic antioxidant analogous to vitamin E) and commercially available tea polyphenols (90% purity) [64]. Fuhalols were the predominant compounds present in the ethyl acetate fraction, followed by phlorethols, fucophlorethols and eckols with varying levels of polymerisation [64]. It is further argued that fuhalol-type phlorotannins possess more vicinal-trihydroxyl, which has the ability to donate H atoms more readily than meta-trihydroxyl elements [64].

As previously described by Leyton et al., phlorotannins (phloroeckol and a tetrameric phloroglucinol, determined by HPLC-ESI-MS) were the most abundant compounds in *Macrocystis pyrifera*, whereas Olate-Gallegos et al., were not able to identify any pholortannins in *Macrocystis integrifolia* (LC-MS/MS); which belongs to the same genus, but is considered an alternative source of flavonoids [65]. Antioxidant activity of *Fucus vesiculosus* extracts were measured using chemical and cell-based assays, suggesting an efficient Fe^2+^ chelating ability and ROS inhibition due to LMW phlorotannins (4–8 PGUs) [167]. It has also been claimed that higher FRAP, DPPH and ORAC values in *D. antarctica* correspond to phlorotannins with 3–8 PGUs, compared to phlorotannins (3–4 PGUs) in *L. spicata* [65,69].

Interestingly, the antioxidant capacity of individual phlorotannins was studied using an online method (UHPLC-DADECD-QTOFMS), exhibiting negative correlations between phlorotannin polymerisation level (molecular weight) and radical scavenging capacity (Hermund et al., 2018). However, these observations pointed out that phlorotannin with five PGUs showed a higher antioxidant capacity than those with four PGUs [116]. This confirms the that the availability of the OH groups is more important in radical scavenging than the degree of polymerisation [116]. It is hypothesised that the structure of large or branched phlorotannins might favour conformations/folding that orientates OH groups towards the inside of the molecules, making them less available to donate H atoms to free radicals, resulting in lower antioxidant activity (Figure 6) [116]. It should be noted that the study suggests the need for further NMR analysis to verify this result [116].

Phenolic acid and flavonoids; In addition to phlorotannins, seaweeds are a rich source of phenolic acid and flavonoid compounds, particularly red and green seaweeds. As shown in Table 4, Enteromorpha (green seaweed), Gracilaria and Callophyllis (red seaweeds) showed the highest scavenging capacity (ABTS) and metal-reducing power (FRAP) due to the hydrolysable polyphenol fractions and the presence of flavonoids, hydroxybenzoic acids and hydroxycinnamic acids [172].

**Figure 10:**
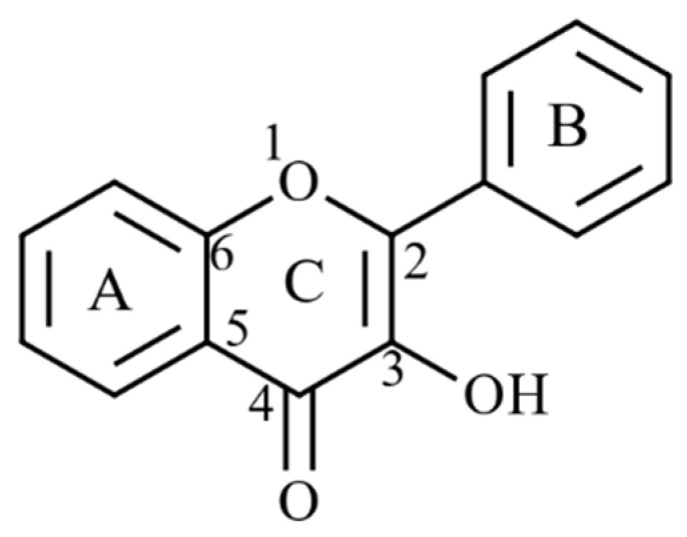
Structural characteristics of the flavonoid responsible for the high radical scavenging potency.

Several brown seaweeds, such as Fucus Sp, *Saccharina japonica* and *Himanthalia elongate*, have phenolic acids (caffeic, gentisic, gallic, vanillic acid, protocatechuic, *p*-hydroxybenzoic, syringic, ferulic, *p*-coumaric, and chlorogenic) and flavonoids (catechin, quercetin, myricetin, and rutin). These have antioxidant properties, including reducing power, radical scavenging activity, lipid oxidation inhibition and metal chelating activity (Table 4). Hydroxyl (OH) groups attached to the benzoic rings serve as H donors in radical scavenging activity. Antioxidant properties of phenolic acids vary depending on the number of OH groups, position, and nature of the substitution of benzoic rings [186]. Substitution of OH groups at the ortho position and the presence of a carboxyl (COOH) in the meta position on benzoic rings favour the formation of highly stable radical intermediates via electron delocalisation. For instance, this occurs in derivatives of hydroxybenzoic acids (gallic, protocatechuic), hydroxycinnamic acids (caffeic) and esters of caffeic and quinic acids (chlorogenic acid) [187]. Benzoic acid with a −CH = CH–COOH moiety enables greater H donating ability in *p*-coumaric, caffeic and ferulic acids [186]. Similarly, flavonoids have become an appealing source of hydroxyl and superoxide radical scavengers (Figure 10). AnOH at the C3 position in the carbon ring favours the antioxidant properties of flavonoids. Moreover, C2–C3 double bonds conjugated with a C4 keto group enhance the radical scavenging potency. Saturation of the C2–C3 double bond also led to a loss of antioxidant capacity [188].

### 7.4. Antioxidant Activity of Seaweed Phenolics in Feed/Ingredient/Animal Model Systems

The antioxidant properties of seaweed compounds have been proven using in vitro assays. However, these assay methods are only an indicator of antioxidant activity. As such, phenolic compounds need to be evaluated in specific model systems to fully understand their antioxidant capability in real food/feed applications [75].

Fish feed: Cian et al. [9] investigated the antioxidant effect, and overall nutritional status of culture juvenile pacú (*Piaractus mesopotamicus*) fed with extruded fish feeds containing 35 g/kg *Pyropia columbina* (red seaweed). After 90 days of dietary treatment, they observed reduced levels of lipid peroxidation, superoxide dismutase and a reduced superoxide dismutase/catalase (SOD/CAT) ratio in the intestine, liver and white muscle of fish compared to fish fed the control diet [9]. In comparison to the control diet (364.6 mg/kg), the experimental diet contained red seaweed rich in phenolic acids (663.7 mg/kg), which were mostly gallic and 4-hydroxybenzoic acids that are known to be compounds with radical scavenging activity [9,153]. In another study, *Carassius auratus* (goldfish) were fed diets containing 50% seaweed biomass (Rhizoclonium: Chaetomorpha: Pithophora: Cladophora; 25% each) for 30 days. This resulted in the fish having significantly higher growth rates, body weight gain, antioxidant levels and skin colouration in comparison to fish fed the control diet [87]. Furthermore, these bioactive compounds minimised ROS formation in fish bodies. Thus, the expression of stress-activated enzymes and lipid peroxidation were minimal and increased fish immunity [87]. The authors reported that both the phenolics and pigments present in seaweed biomass could have caused the beneficial effects in this experiment [87].

Fish oil: Accelerated stability tests using ethanolic extracts of *P. fucoides*, *C. crispes* and *F. distichus* significantly increased the oxidative stability of fish oil compared to fish oil without antioxidants. None of the ethanolic extracts (1000 µg/mL in oil) were as effective as BHT (200 µg/mL in oil) in protecting the fish oil [98]. In contrast, phlorotannin extracts from the brown seaweed *Sargassum kjellmanianum* have been shown to reduce the rancidification of fish oil, where the antioxidant activity of the phlorotannin extract was 2.6 times higher than for BHT [189]. Another study showed that the antioxidant activity of 50% *v/v* ethanolic extract of *Polysiphonia fucoides* (red seaweed) in a 5% fish oil-in-water emulsion was similar to that of BHT [190]. A modified HPLC analysis was performed to quantitatively identify active molecules in the seaweed extracts, suggesting that the phenolic fraction has a higher antioxidant potential than low-molecular-weight, protein-rich, and polysaccharide-rich fractions [190].

Animal models (in vivo): Phlorotannin extracted from *Ecklonia cava* was found to be a therapeutic agent preventing disease associated with AAPH-induced oxidative stress in zebrafish (*Danio rerio*) embryos [191]. Also, phloroglucinols, dieckol, eckol, triphloroethol A and eckstolonol isolated from the seaweed extract exhibited intracellular ROS formation, inhibition of AAPH-induced cell death and lipid peroxidation [191]. Work reported by Kim et al. [192], the phlorotannin extract of *Ecklonia cava* possessed phloroeckol, 6,6-bieckol, phlorofucofuroeckol, dieckol, which inhibited hyperglycemia-stimulated oxidative stress and cell death in zebrafish. Surprisingly, dieckol reportedly reduced nitric oxide (NO), ROS, lipid peroxidation and cell death.

## 8. Seaweed Phenolics as Crosslinkers in Food Delivery Systems (Microencapsulation)

### 8.1. Seaweed as a Source of Biomaterials for Microencapsulation

A range of molecules derived from seaweeds has been applied as ingredients in microencapsulation [193,194]. These compounds can either be structural elements in the shell or provide bioactivity/functionality in the core material of the microcapsules. For example, brown seaweed extracts from *Gracilaria foliifera* and *Sargassum longifolium*, were identified as good sources of antioxidants and antibacterial compounds, and the encapsulation of these extracts in the form of beads showed a significantly high survival rate of tested aquatic species (*O. mossambicus* against *A. salmonicida* infection) [125].

Seaweed polysaccharides are good sources of antioxidants that also possess immune-stimulating, antiviral and antibacterial properties [32]. The prebiotic effect of polysaccharides from *Gracilaria folifera* (red seaweed) was studied by Hindu et al. [195] via feeding trials in *Aeromonas hydrophila*-infected freshwater prawns (*Macrobrachium rosenbergii*) using a microencapsulated probiotic-seaweed polysaccharide diet. The results suggested that a microencapsulated diet with probiotics and seaweed polysaccharides enhanced the antioxidant activity, survival, and immune response in infected *M. rosenbergii* when compared to commercially fed prawns [195]. This emphasises the prebiotic potential of encapsulated seaweed polysaccharides on the performance of probiotic bacteria as a dietary treatment against *A. hydrophila* infection.

Seaweed pigments also possess a range of bio-functionality but are considered an underutilised resource [196]. Seaweed pigments, including chlorophylls and carotenoids, are by-products of alginate production, which are typically bleached and discarded as waste products during the manufacturing process [197]. Seaweed pigments readily oxidise upon exposure to light, heat, and chemical treatments [197]. A previous study encapsulated seaweed pigments, particularly α-and trans-fucoxanthin (a major carotenoid found in brown seaweed), using maltodextrin and Tween-80 matrices and confirmed their stability against photochemical and oxidative degradation [197,198]. The solubility of carotenoids can be enhanced using microencapsulation as they are generally hydrophobic and lipophilic in nature [197].

Seaweed-derived sulphated polysaccharides, namely fucoidan, carrageenan and ulvan, have been extensively studied as drug delivery carriers in the biopharmaceutical industry because of their advantageous pharmacokinetics and oxidative and thermal stability [193]. In particular, the presence of glycosidic bonds, hydroxyl groups and negatively charged sulphate groups in seaweed polysaccharides contribute to biodegradability and polyelectrolyte behaviour in biomaterials [199]. For instance, fucoidan from *F. vesiculosus* has been used as a shell material to encapsulate Ofloxacin (OFL), a broad-spectrum antibiotic, to overcome poor solubility in serum [200]. The fucoidan microspheres resulted in enhanced drug encapsulation efficiency compared to chitosan microspheres [200]. Fucoidan has also been used as a crosslinking agent to crosslink protein (bovine serum albumin)—chitosan microcapsules to create slow-release macromolecular drugs such as protein and peptides [201].

### 8.2. Microencapsulated Oil in Aquafeed

Dietary fat, including fish oil, is one of the major constituents found in aquafeed, which provides energy and essential fatty acids for the growth and development of aquaculture species [202]. Fish oil is rich in long-chain PUFAs and is readily oxidised during processing and storage. For this reason, it requires protection for long-term preservation [203]. Microencapsulation is a viable solution to protect fish oil in aquafeed, as this process forms a physical barrier called a “shell” around the “core” oil, which transforms liquid fat into free-flowing dry powder [204,205].

To date, a variety of hydrophobic bio-functional substances have been subjected to microencapsulation, including plant extracts, essential oils, marine oils, pigments, flavours, nutraceuticals, and fat-soluble vitamins [204,205]. Anchovy oil was microencapsulated using gelatin-sodium hexametaphosphate complex coacervation, achieving a 53% payload (oil to capsule weight ratio), 98% encapsulation efficiency 98% encapsulation yield [205,206]. Compared to liquid dietary fat, microencapsulated fish oil has a range of benefits, such as improved oxidative stability, masking fishy odours and flavours, controlled release rate, prevention of co-reactions with other compounds, and easy handling due to its powder form [205]. Considering this, Ma et al. [204] compared the impact of fish oil supplements in both liquid and microencapsulated forms in Nile tilapia diets, revealing that microencapsulated oil enhanced fish immunity via enhancing gut microflora and structure.

### 8.3. Phenolics as Cross-Linkers in Complex Coacervation

In terms of high efficiency (>99.9%), payload (>50%) and controlled release of bioactive ingredients, microencapsulation that uses complex coacervation is a superior method compared to other techniques such as spray drying, freeze-drying, extrusion, fluidised bed coating and melt injection [205]. Complex coacervation is conducted via a multi-step process following emulsification, coacervation, hardening and cross-linking [205]. First, the core material is emulsified in a continuous aqueous phase containing two more oppositely charged polymers (usually protein and polysaccharides) at a pre-defined temperature and pH (which is above the isoelectric point of the protein). Secondly, the pH of the emulsion is decreased below the protein’s isoelectric point to form insoluble free coacervates via electrostatic binding between the oppositely charged polymers, which triggers phase separation. Subsequently, insoluble complex coacervates migrate to the oil/water surface and form a visualised shell (aggregation) around emulsion droplets. Finally, microcapsules are cross-linked chemically or enzymatically to stabilise the shell structure, resulting in microcapsules that can be further subjected to drying (e.g., spray drying, freeze-drying) to obtain a free-flowing dried powder. Cross-linking is an important step in microencapsulation as the binary coacervate complex is highly unstable under pH and/or temperature variations, influencing rheological properties and stability [207].

Chemical cross-linkers, such as glutaraldehyde and formaldehyde, are cheap and effective choice but are linked to carcinogenicity and genetic toxicity. Today, safer and natural substitutes are used, such as transglutaminase. However, they are expensive, and the enzymatic process is relatively slow [208,209]. As reported previously, polyphenols are believed to be a non-toxic and natural solution for cross-linking, which are also economically viable [210]. For instance, a comparative study evaluating polyphenols (360 μg polyphenols/g gelatin) and genipin (300 mg genipin/g dry gelatin) as cross-linkers in hydrogel formation demonstrated that polyphenols had high efficacy in terms of thermal stability and gel strength.

Tannins are high molecular weight phenolic compounds that occur naturally in higher plants and possess multiple functional groups; −OH and −COOH [211]. As shown in Table 5, tannic acids have been widely used to study cross-linking properties among other phenolic groups, such as phenolic acids (caffeic, ferulic and chlorogenic) and flavonoids (rutin) with different protein solutions (e.g., gelatin, casein and whey protein isolate). Protein–polyphenol cross-links are formed through hydrogen, hydrophobic, and covalent bonds [209], which permits the creation of stable cross-links between proteins and carbohydrates [211]. Phlorotannins are similar to terrestrial tannins, sharing similar structural characteristics [211], thus emphasising the potential of seaweed-derived phenolics as potential cross-linking agents in ingredient delivery and hydrogel applications. Nevertheless, to date little research has been undertaken to explore seaweed phlorotannin in complex gel systems.

Muhoza et al. [209] determined the thermal, rheological, and morphological characteristics of gelatin-high methyl pectin coacervates (G-HMP) cross-linked by tannic acid (TA) and their use for the encapsulation of peppermint oil. They found that the TA cross-linking significantly altered the secondary structure of gelatin, resulting in larger coacervate aggregates with rough and irregular shapes, whereas non-cross-linked G-HMP coacervates were found to be smooth and spherical in shape [209]. During hardening, coacervates enlarged from 18 to 36 μm due to the intermolecular bonds formed by TA cross-linking, which improved gelling and melting points, elastic behaviour, and thermal stability [209]. With such benefits, peppermint oil microcapsules were quite large (47 μm) with high encapsulation efficacy (75%) and thermal stability [209]. Moreover, several studies were conducted using oxidised (OX-TA) and non-oxidised (NO-TA) forms of tannic acid to cross-link coacervates in two model systems (hydrogel and microcapsules). NO-TA provided stronger intermolecular connectivity via hydrogen bonding and enhanced mechanical and gelling properties compared to OX-TA cross-linked coacervates [212]. In contrast, Mohseni et al. [213] found improved oxidative stability of flaxseed oil microcapsules cross-linked with OX-TA. In addition to tannic acids, coffee and white grape juice have also been shown to have sufficient phenolic content, which can be directly used in cross-link formation without isolating active compounds [214]. Cross-linking properties of phenolic compounds and their properties in a protein–polyphenol complex (e.g., microencapsulation, hydrogels, emulsions) have been summarized in Table 5.

### 8.4. Protein–Polyphenol Interactions

In general, hydroxyl groups and aromatic rings in polyphenols readily interact with the amine groups of proteins via covalent and non-covalent bonds at a wider pH range (pH 3.2 and 5.2) [219]. It has been previously reported that sunflower proteins can be cross-linked by chlorogenic acid through covalent and hydrogen bonds that exist between cysteine and lysine amino acid residues (nucleophilic) and oxidised forms of phenolic acid [220]. Covalent conjugates between protein and phenolic molecules are stronger and more stable than those formed through non-covalent bonds; thus, the covalent bond formation is preferred in their use across various applications [64].

**Figure 11:**
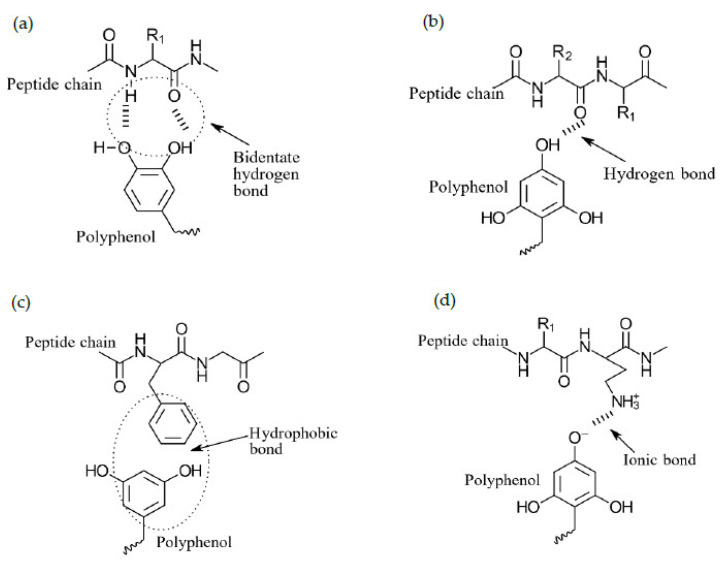
Protein and polyphenol interactions (cross-links) via non-covalent bonds (**a**) bidentate hydrogen bond (**b**) hydrogen bond (**c**) hydrophobic bond (**d**) ionic bond.

Non-covalent interactions (Figure 11)**:** Hydrogen bonding and hydrophobic interactions are the most common non-covalent bonds between protein–polyphenols conjugates and are usually reversible and lower-energy than covalent interactions [221]. Ionic bonds exist but only play minor roles [222]. Hydroxyl groups in polyphenol compounds contribute to hydrogen bonding via interacting with the −OH or −NH_2_ groups on the sidechains of proteins [223]. Moreover, aromatic phenolics are excellent hydrogen donors and readily interact with the carbonyl groups (−C=O) of protein molecules via H-bonds [224]. Previously, FTIR analyses and isothermal titration calorimetry (ITC) measurements have shown that phenolic-protein cross-links were mainly driven by non-covalent bonds (e.g., H-bonds) [210,215].

**Figure 12:**
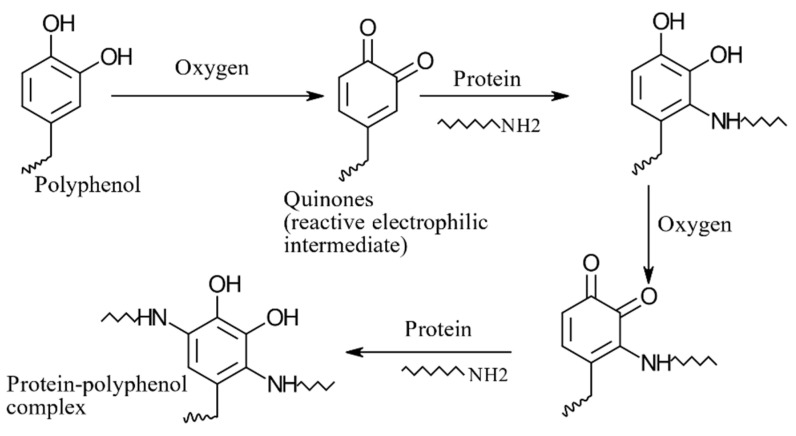
Protein and polyphenol (oxidized form) interactions via covalent bonds.

Covalent interactions (Figure 12)**:** Covalent cross-links can be achieved via either non-enzymatic or enzymatic approaches. Both oxidised and non-oxidised forms of TAs are used to enhance the gelling and emulsifying ability of proteins via covalent bonds [212]. Non-enzymatic approaches use alkaline pH (9.0) in the presence of oxygen, where phenols are transformed into semi-quinone radicals and further into quinones (reactive electrophilic intermediates)[89,225]. Quinones are readily attacked by nucleophilic amino acid side chains (lysine, methionine, cysteine, tryptophan) via C-S, C-N covalent cross-links, resulting in robust protein–polyphenol conjugates [226]. Enzymatic cross-linking is an environmentally friendly approach performed under mild reaction conditions (40 °C). Covalent cross-links were formed between whey protein isolate (WPI) and β-lactoglobulin coacervates using transglutaminase under mild reaction conditions [227].

### 8.5. Factors Effecting Protein–Polyphenol Interactions

Several studies have investigated the effect of protein–polyphenol cross-linking on the stability of the encapsulants. Some parameters that must be carefully considered include phenolic structure, protein type and size, amino acid composition, protein concentration, temperature and pH. These aspects have been previously discussed in detail [220,225].

Structure of phenolic compounds: Properties of phenolic compounds must be taken into account for protein–phenolic interactions; these include molecular weight, hydroxylation, hydrogenation, methylation and glycosylation [225]. The number of cross-linking sites increases with increasing numbers of hydroxyl groups in phenolic molecules, for example, epigallocatechin gallate > epicatechin gallate > epicatechin > catechin [218]. Binding affinity between protein (β-casein) and polyphenol showed positive correlations with the molecular mass of polyphenols [228]. Black tea polyphenols, including thearubigin and theaflavin, showed better cross-linking with milk proteins due to the higher degree of catechin monomer polymerisation. However, milk and tea cross-linking diminish their radical scavenging capacities, as free hydroxyl groups in catechin are reduced by cross-links [229].

Bartolomé et al. [220] studied phenolic structure and how the presence of other phenolic compounds in the reaction media influences the extent of interactions between BSA and LMW phenols. Among the tested compounds (catechin, p-coumaric acid, caffeic acid, p-hydroxybenzoic acid and protocatechuic acid), the highest protein binding affinity was observed with protocatechuic, caffeic and protocatechuic acids while no interactions were observed between BSA and p-hydroxybenzoic acid [220]. The presence of o-dihydroxy groups in caffeic and protocatechuic acids leads to the bidentate hydrogen bond between phenolic OH and peptide bonds (N-H and C=O groups, Figure 11) which are more stable than single H bonds [220,230].

Modifications of flavonoid structure occur in plants via methylation, hydroxylation, glycosylation and acylation. These modifications occur on the hydroxyl groups in the A, B and C-rings in flavonoid compounds [231]. According to Xiao et al. [230], methylation of flavonoids has little influence on milk protein binding affinity, whereas hydroxylation of flavanone ring A significantly enhanced the degree of milk protein cross-linking. However, the hydroxylation of the C ring of flavones and A ring of isoflavones showed no impact on cross-linking [230]. Furthermore, glycosylation (substitute of OH groups by glycoside) of flavonoids weakened milk protein binding affinity due to steric hindrance, while hydrogenation of C2 = C3 bonds in many flavonoids decreased the protein binding ability of BSA [230]. Moreover, the presence of catechins significantly enhanced the binding of milk protein [230].

Temperature: This is one of the most important cross-linking parameters as it affects hydrogen bonding and hydrophobic interactions. Many examples in the literature show that the ability of phenolic compounds to bind protein increases with temperature via hydrophobic and H bonding, as heating can expose previously buried hydrophobic domains of peptide chains [89]. For example, caffeic acid cross-linked with the amino acid residuals of sunflower seed proteins via hydrogen and ionic bonds between 10 and 45 °C. Increasing the temperature further to 90 °C caused the binding affinity of phenolics for proteins to decrease due to an increase in hydrophobic interactions [232]. Some contradictory results exist in the literature that suggest temperature increases negatively impact cross-linking. For instance, increasing temperature from 30 to 45 °C significantly reduced binding between 5-O-caffeoylquinic acid and sunflower seed protein (11S), whilst binding at 55 °C was abolished [233]. This highlights the importance of temperature control in cross-linking, as conformational changes or partial denaturation of proteins can alter binding site availability for phenolic compounds.

pH: At pH values below the isoelectric point of proteins, such as pH 0.3–3.1, proteins become more dissociated, and more binding sites are available, resulting in more extensive cross-linking [225]. Non-covalent bonds preferentially form at pH ≤ 7, while alkaline pH causes autoxidation of monomeric phenolic acids, which leads to the formation of quinones/radicals and subsequently produces covalent bonds [234].

Protein type and concentration: The protein type and molar ratio of protein to phenolic are important factors in cross-linking [234]. Cross-links occur either hydrophilically or hydrophobically, depending on the number of binding sites available on a particular protein [225].

## 9. Safety, Legal and Ethical Aspects of Seaweed in Aqua Feed Products

Currently, there are no known limits or legitimate concerns, regarding possible harmful effects to either animal or human health, for the use of seaweed phenolic compounds in aquaculture. For centuries, seaweeds have generally been considered safe to consume in relatively large quantities. However, in 1990 France was the first European country which to establish certain rules and regulations for seaweed consumption as a non-traditional food substance [235]. Nowadays, seaweeds are considered a “novel food” under European regulations (Regulation (EU) 2017/2470), and up to 22 seaweed species (European and imported seaweeds) had been listed under this category at the end of 2020 [236]. Although seaweeds do not produce endogenous toxins, the presence of high levels of toxic contaminants, such as residual pesticides, minerals, and heavy metals (cadmium, mercury, tin, lead, etc.) likely limit their applications as human food and animal feed ingredients [237].

Food Standards Australia New Zealand (FSANZ) monitors inorganic arsenic and iodine levels in seaweed species and seaweed products [238]. Arsenic exists in both organic and inorganic forms, but inorganic arsenic is highly toxic relative to the organic form [238]. These elements naturally occur in water and soil, making their presence in food unavoidable, but they can also bioaccumulate through contamination leading to elevated levels [238]. According to FSANZ, a 1 mg/kg limit applies to seaweed, molluscs, fish and crustaceans, with levels beyond 2 mg/kg not allowed [238]. These regulatory limits vary between countries and jurisdictional organisations as the analytical methods are unique to different authorities. For instance, the permitted limit of inorganic arsenic in the European Commission (EC) is higher than the limits of FSANZ [238].

According to a recent dietary survey, certain seaweed species and seaweed products are considered risk foods as they do not comply with the limits of the Food Standard Code. For example, *Sargassum fusiforme*, due to naturally occurring arsenic (Hijiki dried, 7.8 mg/kg) is non-compliant but still regularly consumed in certain cultures [239]. Roleda et al. [237] investigated the variation of polyphenol and heavy metal (Cd, As, Pb and Hg) concentration in *Laminaria saccharina* (brown seaweed), *Alaria esculenta* (brown seaweed) and *Palmaria palmata* (red seaweed). The highest phenolic content was recorded in *A. esculenta* followed by *L. saccharina* and *P. palmata*. All three samples were suggested as good sources of antioxidants whilst the concentration of heavy metals remained below the maximum residual limits (MRLs) according to European Commission and French recommendations [237]. Heavy metal concentration can vary with seaweed type and harvest location, with the effects of seasonal variation, thought to be minimal [237]. Regarding iodine, the survey revealed that most seaweeds are within safe levels, except Laminariaceae sp. (Kombu). According to (EC) NO 396/2005, the MRLs of pesticides in seaweed have been established only for human consumption, which is not applicable for feed purposes [161]. Proper pre-treatment, extraction and purification methods could be used to eliminate toxic compounds from seaweed extracts.

Apart from safety regulations, there are also ethical aspects to be considered for wild seaweed harvesting, such as loss of species, habitat diversity, over-harvesting, increased sedimentation, eutrophication, destruction of coral reefs and conflicts with coastal communities. Today, over 800,000 t wild seaweed are harvested annually from natural beds by 32 countries, which constitutes an integral role and a part of the cultural identity of those coastal communities [240]. With the commercial demand, it is crucial to ensure sustainable seaweed harvesting and effective management practices (e.g., processing, packaging and transportation) by coastal communities. For example, environmentally damaging seaweed harvest techniques must be replaced by well-maintained harvesting tools with a capacity for further expansion. Moreover, harvesters require to determine the quantity of biomass and time necessary to successfully complete the task prior to purchasing the seaweed beds from landowners and initiating harvesting [240]. Collaborative efforts of governments, international organizations, the scientific community, the industry, stakeholders and civil societies need to be proactive in sustainable seaweed harvesting and management.

## 10. Conclusions & Future Prospects

Synthetic additives are essential components in aquafeed formulations; however, many have been reported to adversely affect the health of aquatic life and humans. These additives are now banned in many regions, jeopardising the long-term sustainability of the global aquaculture industry. As such, establishing safer natural feed additives from sustainable sources is now a matter of great interest. Seaweed phenolics are a highly heterogeneous group of compounds with potential therapeutic, biological and functional activities against fish pathogens (*Vibrio* sp., *Aeromonas* sp.) and foodborne pathogens (*E. Coli*, *Salmonella* sp., *Streptococcus* sp.). Antioxidant properties are by far the most studied bioactivities found in seaweed phenolics, and close attention must be taken when using specific model systems (e.g., fish feed, fish oil, and animal models in vivo) to fully understand the antioxidant capability of various seaweed phenolics in real food/feed applications. Because the majority of antioxidant studies were conducted with non-purified seaweed extracts, an understanding of the antioxidant efficacy of phenolic compounds (particularly in relation to their molecular structures) is lacking and requires further research.

The functional groups in phenolics readily interact with the amine groups of proteins via covalent and non-covalent bonds, giving improved rheological, mechanical strength and stability, which are potentially useful in the formation of successful hydrogels edible films and microcapsules systems. However, the lack of information on the crosslinking ability of seaweed phenolics does not allow in-depth assumptions, particularly via covalent and non-covalent bonds. Methodologies that are able to quantify these aspects in terms of the stability and mechanical and rheological properties of final products are recommended for future studies. Conventional extraction, separation and purification steps are established for isolating phenolic compounds; however, much work remains in the development of industrially relevant novel and green phenolic processing methods. Scalable, high-throughput technologies will be required to explore the supplementation of aquafeeds with sufficient seaweed phenolics and maximise their benefits. Developing seaweed phenolics in food, feed and pharmaceutical applications as a bioactive and functional additive requires further consideration of safety and legal aspects. Finally, more attention is required to implement sustainable seaweed harvesting and management approaches to prevent overexploitation of natural seaweed beds while ensuring constant seaweed supply to fulfil global demand. The expansion of the seaweed industry is potentially improved with sea/inland farming, sustainable harvesting and processing techniques, strain improvement, diversification of species and the establishment of relevant policy.

## Figures and Tables

**Table 1:** Antibiotic and antimicrobial properties of seaweed phenolic compounds against common pathogens found in aquaculture.

Seaweed Species	Seaweed Extract	Polyphenol Content/ Active Compounds	Pathogenic Species	Antibiotic and Antimicrobial Properties	Reference
*Gracilaria folifera* (Red seaweed), *Sargassum longifolium* (Brown seaweed)	Ethanolic and aqueous extract (250 mg/L).	TPC; 5.2 mg GAE/g, and 2.8 mg GAE/g in ethanolic extract (Folin–Ciocalteu Assay) TFC; 9.3 mg QE/g and 8 mg QE/g in ethanolic extract	*Aeromonas salmonicida*	Antibacterial activity Relative percentage of survival; 90% till 120 h	[119]
*Sargassum wightii* (Brown seaweed), *Ulva lacta* (Green seaweed), *Padina tetramatica* (Brown seaweed)	Methanolic extract Diethyl ether extract Methanolic extract	*Sargassum wightii* exhibited high phenolic content	*Vibrio alginolyticus* (VA09), fish pathogenic bacteria	Antimicrobial activity; Minimum inhibitory concentration (MIC) of extracts were 25 mg/mL, 50 mg/mL and 50 mg/mL respectively	[120]
*Sargassum muticum* (Brown seaweed)	Crude acetone-water seaweed extracts and purified (SPE) methanol-water seaweed fraction	TPC; 17% DW_fraction_ in crude extract and 1.45% DW_seaweeds_ after SPE extract Phlorotannins; phlorethol (^1^H NMR and 2D NMR)	*Vibrio aestuarianus* *Vibrio anguillarum* *Vibrio parahaemolyticus*	Antibacterial activity; Crude extract (>50% bacterial growth inhibition) >> Purified fraction	[86]
*Chaetomorpha antennina* (Green seaweed)	Ethanolic extract	TPC; 180 mg GAE/g DW TFC; 79.6 mg QE/g DW	*Vibrio parahaemolyticus* (shrimp pathogen)	Antibacterial activity Extracts of 50 μL, 100 μL, 150 μL, 200 μL showed zone of inhibition 17 mm, 21 mm, 28 mm, and 36 mm respectively.	[121]
*Sargassum thunbergii* (Brown seaweed)	Ethanolic extract fraction	Low molecular weight phlorotannins (LMPs)	*Vibrio parahaemolyticus* (marine bacterium associated with human infection)	Antibacterial property Growth curve; LMPs (900 μg/mL) prevented cell division at logarithmic growth phase *vs* control group grew towards the stationary phase	[122]
*Ecklonia Arborea* (Brown seaweed)	Crude extract (CE) and phlorotannin-enriched ethyl acetate fraction (EPE).	Phlorotannins; Eckol (5.23 mg/g) and dieckol (1.67 mg/g) (HPLC/MS-TOF)	*Vibrio parahaemolyticus* (Acute hepatopancreatic necrosis disease (AHPND) of shrimps	EPE bactericidal activity 4.6-fold higher than CE. Minimum bactericidal Concentration; CE (3500 μg/mL) vs. EPE (750 μg/mL)	[123]
*Cladophora glomerate,* *Rhizoclonium crassipellitum, Chaetomorpha aerea, Pithophora cleveana*; (Green seaweed)	50% seaweed biomass-added to fish feed Biomass includes each 25% for algal biomass	TPC; max 52.55 ± 0.01 mg GAE/g DW in algal biomass TFC; max 71.8 ± 0.21 mg QE/g DW in algal biomass	Fed to *Carassius auratus* (goldfish)	Skin pigmentation, Growth rate and antioxidant activities; 1.44–4-folds increase compared to the control group	[87]
*Padina australis* Hauck (Brown seaweed)	Ethyl acetate fraction (EAF)	Presence of tannin, steroid, phenolic, alkaloid and terpenoid compounds in EAF	*Vibrio harveyi, Vibrio parahaemolyticus*, and *Aeromonas hydrophilla* Shrimp pathogenic bacteria	Antimicrobial activity Average zone of inhibition for *Vibrio harveyi*, *Vibrio parahaemolyticus* and *Aeromonas hydrophilla* (EAF; 1.76 mm/ 2.3 mm/ 4.43 mm vs. Ciprofloxacin; 9.3 mm/ 6 mm/ 9.41)	[124]
*Gracilaria foliifera* (Red seaweed), *Sargassum longifolium* (Brown seaweed)	Purified ethanolic fraction of seaweed	TPC; 18.42 mg/g GAE and 14. 71 mg/g GAE TFC; 14.71 mg/g QE and 17.21 mg/g QE	*Aeromonas salmonicida* infection in *Oreochromis mossambicus* (Mozambique tilapia)	Antibiotic activity Minimal inhibitory concentration of *G. folifera*, *S. lonfifolium*, negative control, positive control (antibiotic) were 15 μg/mL, 20 μg/mL, 0 and 15 mg/mL respectively.	[125]
*Sargassum thunbergii* (Brown seaweed)	Purified ethanolic fraction	Low molecular weight phlorotannin (LMPs); 900 μg/mL	*Vibrio parahaemolyticus*	Antibacterial activity Growth curve; LMPs (inhibited thalli growth at logarithmic phase) vs. control (started growth in logarithmic phase); Membrane permeability- protein content of culture media; LMPs (256.79 μg/mL) vs. control (47.73 μg/mL)	[122]

TPC—Total Phenolic Content, TFC—Total Flavonoid Content, GAE—Gallic acid equivalence, QE—Quercetin equivalence, SPE—Solid Phase Extraction, DW—Dry Weight. Search keywords: seaweed phenolics, phlorotannins, bioactivity, antibacterial, antifungal, antimicrobial, aquaculture pathogens, shrimp pathogen; Years of searching: 2010–2022

**Table 2:** Antimicrobial properties of seaweed phenolic compounds against foodborne pathogens.

Seaweed Species	Seaweed Extract	Polyphenol Content/Active Compounds	Antimicrobial Properties	Reference
*Ascophyllum nodosum,* *Fucus serratus* (Brown seaweed)	Phlorotannin extracts	TPC, *A. nodosum*; 37.35 mg/g (^1^H NMR) and 30.68 PGE/g (FC assay) TPC, *F. serratus*; 17.00 mg/g (^1^H NMR) and 36.68 PGE/g	Antimicrobial activity against *Escherichia coli*, *Salmonella agona*, and *Streptococcus suis* (foodborne pathogens) Minimum inhibitory Concentration; *A. nodosum* (1.56–0.78 mg/mL) and *F. serratus* (3.13mg/mL) and Minimum Bactericidal Concentration; *A. nodosum* (3.125–1.56 mg/mL) and *F. serratus* (6.25 mg/mL)	[126]
*Himanthalia elongate* (Brown seaweed)	Dried methanolic extracts	TPC; 151.3 mg GAE/g TFC; 42.5 mg QE/g Total tannin; 38.34 mg CE/g	Antimicrobial activity against *L. monocytogenes, S. abony* and *E. faecalis*, and *P. aeruginosa* (food borne and food spoilage bacteria); *H. elongate* extract, Sodium benzoate, Sodium nitrite; up to 100%, 99–89% and 98–93% inhibition respectively	[127]
*Padina boergesenii* (Brown seaweed)	Polyphenol extract	NA	Antibacterial activity against antibiotic resistant *E. coli* strains; most of the bacteria inhibited within 256 µg/mL concentration	[128]
*Turbinaria ornate* *Sargassum wightii* (Brown seaweed)	Methanolic extract	TPC; 43.72 and 35.98 mg GAE/g extract respectively	Antibacterial activities against *Bacillus subtilis, E. coli, Shigella flexnerii* and *Staphylococcus aureus* Zone of inhibition (mm); *T. ornate*, *S. wightii* and standard were max 20 mm, 18 mm and 28 mm respectively	[129]
*Sargassum tenerrimum* and *Turbinaria ornate* (Brown seaweed)	Chloroform extract	TPC; 3.598 mg/g and TFC; ~0.15 mg/g TPC; ~2 mg/g and TFC; ~0.1mg/g	Antifungal activity against *Aspergillus niger* and *Penicillium janthinellum; S. tenerrimum* 100 µL (20 mm,14 mm), *T. ornate* 100 µL (13 mm, 16 mm), Fluconazole 10 mcg (10 mm, 12 mm), Ketoconazole 10 mcg (17 mm, 20 mm), Amphotericin B 20 mcg (18 mm, 19 mm), Negative Control (8 mm, 9 mm)	[130]
*Padina Pavonica* (Brown seaweed)	Ethyl acetate fraction	TPC; 8.98 GAE/g	Antifungal activity against *Candida glabrata* (diameter of inhibition = 16 mm) and *Candida krusei* (diameter of inhibition = 14 mm)	[131]
*Himanthalia elongate* (Brown seaweed)	Ethanolic extract	TPC; 18.79 mg GAE/g	Antimicrobial activity against *Salmonella spp.* *Listeria monocytogenes* *Escherichia coli* *Staphylococcus aureus* *Bacillus cereus*	[132]

NA—Not available, TPC—Total Phenolic Content, TFC—Total Flavonoid Content, GAE—Gallic acid equivalence, QE—Quercetin equivalence, CE—Catechin equivalents, PGE—Phloroglucinol equivalents, DW—Dry Weight. Search keywords: seaweed phenolics, phlorotannins, bioactivity, antibacterial, antifungal, antimicrobial, foodborne pathogens, food spoilage pathogens; Years of searching: 2010–2022.

**Table 3:** Seaweed phenolics as potential veterinary medicine in clinical studies.

Seaweed Species	Seaweed Extract	Polyphenol Content/ Active Compounds	Tested Species/Cell Line	Dosage	Therapeutic Properties	Reference
*Eisenia bicyclis* (Brown seaweed)	Ethyl acetate fraction	Phlorofucofuroeckol A and dioxinodehydroeckol Dieckol and 7-phloroeckol Phloroglucinol	RAW 264.7 murine macrophages cells	>10 µg/mL >50 µg/mL >100 µg/mL	Cytotoxicity Prevent inflammatory and oxidative stress-related diseases	[133]
*Ecklonia radiata* (Brown seaweed)	Ethyl acetate fraction	TPhC; 619 PGE mg/g (eckol-type phlorotannins)	Neuronal PC-12 cell line	100 µg/mL	Neuroprotective activity against the neurotoxic amyloid β protein (Aβ_1–42_)	[134]
*Halimeda opuntia* (Green seaweed)	Methanolic extract	TPC; 55.04 mg GAE/g of extract	MCF-7 & 3T3 cell lines	25.14 µg/mL and 65 µg/mL	Cytotoxicity	[135]
*Ascophyllum nodosum* (Brown seaweed)	Ethanolic extract	High molecular weight fraction (>10 KDa) TPhC; 938.2 µg PGE/mg hydroxytrifuhalol A, C-O-C dimer of phloroglucinol, dimer diphlorethol, difucol and 7-hydroxyeckol	HT-29 cell culture	250 μg/mL	Effect of simulated gastrointestinal digestion and fermentation	[136]
*Sargassum muticum* (Brown Seaweed)	Methanolic extract	TPC; 78.95 ± 4.33 mg GAE/ 100 g dried plant	MCF-7 and MDA-MB-231 breast cancer cell lines	22 μg/mL and 55 μg/mL	Antioxidant, Antiproliferative, and Antiangiogenesis Effects	[137]
*Gracilaria* *Fisheri* (Red Seaweed)	Ethanolic extract	NA	virulent strain of *Vibrio* *harveyi*	MIC; 90 µg/mL	Immunostimulant and anti-bacterial activity	[138]
*Sargassum horneri* (Turner) C. Agardh (Brown Seaweed)	Ethanolic extract	NA	RAW 264.7 murine macrophage cell line	200 µg/mL	Anti-inflammatory activity	[139]
*Eisenia arborea* (Brown Seaweed)	Methanol-chloroform extract	Phlorotannins (eckol, 8,8′-bieckol, phlorofucofuroeckol (PFF)- A and PFF-B	ICR mice	0.1 mg/mouse	Anti-allergic and anti-inflammatory effects	[140]
NA	NA	Commercially purchased Dieckol	Rats	20 mg/kg bwt	Anticancer, anti-inflammatory, and anti-cell proliferative effects	[141]
*Agarum cribrosum* (Brown Seaweed)	Ethyl acetate fraction	Trifuhalol A	RAW 264.7 cells	Hyaluronidase inhibitory activity (200–1000 µg/mL) Proliferation, NO production, cytokines mRNA expression (5–20 µg/mL)	Anti-inflammatory Activity	[142]
*Ecklonia cava* (Brown Seaweed)	Phlorotannin-rich extract	Dieckol 98% phloroglucinol equivalent	Mice	50– 100 mg/kg/d	Prevent lipopolysaccharide (LPS)-induced septic shock	[143]

NA—Not available, TPC—Total Phenolic Content, TPhC—Total Phlorotannin Content, GAE—Gallic acid equivalence, PGE—Phloroglucinol equivalents, DW—Dry Weight. Search keywords: seaweed phenolics, phlorotannins, bioactivity, veterinary medicine, aquaculture, therapeutic, cytotoxicity, anti-Inflammatory, immunostimulant, neuroprotective, clinical studies; Years of searching: 2010–2022

**Table 4:** Antioxidant properties of seaweed phenolic compounds.

Seaweed Species	Tested Sample	Phenolic Content/Active Compounds	In Vitro Antioxidant Activities	Application	Reference
*F. vesiculosus, F. serratus, F. distichus, F. spiralis* (Brown seaweed)	Aqueous extracts	High levels of caffeic and gentisic acid and relatively high levels of gallic and vanillic acid	DPPH, RAP, oxidation inhibition in liposome model system.	Preserving fish oil	[98]
*Gelidiella acerosa* (Red seaweed)	Methanolic extract	TPC; 0.616 g/g GE	DPPH, Inhibition of lipid peroxidation, nitric oxide radical scavenging activity, hydrogen peroxide scavenging activity, RAP	Food preservative and therapeutic agent for oxidative stress-related disorders	[166]
*Fucus Vesiculosus*, *Bifurcaria bifurcate*, *Ascophyllum nodosum* (Brown seaweeds)	Aqueous extract	TPhC (FVE); 1.15 g PGE/100 g extract TPhC (BBE); 1.99 g PGE/100 g extract	ABTS, DPPH, ORAC and FRAP	Oil stabilizers in canola oil (500 ppm seaweed extract > 50 ppm BHT)	[154]
*Ecklonia radiata* (Brown seaweed)	Seaweed extract	TPhC; 4.4 g (PGE) 100 g^−1^ (DW)	FRAP, ORAC	Natural antioxidant and functional food ingredient	[93]
*Fucus vesiculosus* (Brown seaweed)	Aqueous seaweed extracts	TPC; 0.26 and 0.30 g PGE/g Phlorotannin-LMW; fucodiphlorethol A and trifucodiplorethol isomers (HPLC-DAD-ESI-MS)	ORAC, DPPH, FCA, ABTS, CAA	Antioxidant potential of enriched convenience cereals	[167]
*Saccharina japonica* (Brown seaweed)	SWE + IL extract	TPC; 39.55 mg PGE/g DW Chlorogenic, Protocatechuic, *p*-Hydroxybenzoic, Gentisic, Caffeic, Gallic, Syringic	DPPH, ABTS, TAC, FRAP	NA	[168]
*Fucus vesiculosus L.* (Brown seaweed)	Methanol/water extracts	TPC; 41.4 gPEGkg^−1^ DM	DPPH, FRAP, inhibition of copper-catalyzed LDL oxidation	NA	[100]
*Bifurcaria bifurcate* (Brown seaweed)	Organic extract (methanol and acetone) and aqueous extract	TPC; 2.0–2.5 g PGE 100 g^−1^	DPPH, Reduction of power	Antioxidant nutraceuticals	[99]
*Durvillaea antarctica,* *Lessonia spicata,* *Macrocystis integrifolia* (Brown seaweed)	Ethanol/water extract	*D. antarctica*; TPC 5 g PGE/kg DW and *L. spicata*; TPC 1.21 g PGE/kg DW; phlorotannins (trimer to tetramer). *M. integrifolia*; TPC 3.7 g PGE/kg DW; flavonoids (glycoside forms)	FRAP, ORAC, DPPH	Food and pharmaceutical applications	[65]
*Hypnea musciformis* (Red seaweed)	Ethyl acetate fraction	TPC; 205.5 mg GAE/g	DPPH, ABTS, FCA, H_2_O_2_ scavenging activity, lipid peroxidation inhibitory activity	Food preservative	[169]
*Ascophyllum Nodosum,* *Fucus vesiculosus,* *Fucus serratus* (Brown seaweed)	Ethanol/water extract	TPC; 21.42 g PGE/100 g extract TPC; 22.71 g PGE/100 g extract TPC; 12.36 g PGE/100 g extract	DPPH *A. Nodosum* *>* *F. Serratus* *>* *F. Vesiculosus*	NA	[101]
*Caulerpa lentillifera*, *C. racemose* (Green seaweeds), *Sargassum polycystum* (Brown seaweed)	Methanolic extract	TPC; 42.85 mg PGE/g DW TPC; 40.36 mg PGE/g DW TPC; 45.16 mg PGE/g DW	TEAC, FRAP	Natural antioxidants	[170]
*Sargassum sp.* (Brown seaweed)	Hot water extract	TPC; 2.4 mg GAE/g DW	DPPH	Functional food ingredient	[96]
*Turbinaria ornate* (Brown seaweed)	Methanolic extract	TPC; 2.07 mg catechin/g DW	ABTS, DPPH, RAP	NA	[171]
*Ulva sp.* (Green seaweed) *Gracilaria chilensis, Callophyllis concepcionensis* (Red seaweeds)	Hot water extracts	TPC; 551.1 mg GAE/100 g DW TPC; 216.4 mg GAE/100 g DW TPC; 218.6 mg GAE/100 g DW (Hydrolyzable polyphenols; hydroxycinnamic acids, hydroxybenzoic acids and flavonols)	ABTS, FRAP	Natural antioxidant	[172]
*Cystoseira trinodis* (Brown seaweed)	Dichloromethane fraction from crude methanolic extract	TPC; 17.30 mg GAE/g of fraction Active compound; phlorotannins	DPPH	NA	[173]
*Sargassum horneri* (Brown seaweed)	Ethanolic extract in SC-CO_2_	TPC; 0.64 ± 0.02 mg GAE/g TFC; 5.57 ± 0.05 mg catechin/g	DPPH, ABTS	Natural antioxidant	[174]
*F. vesiculosus, F. serratus, A. nodosum* (Brown seaweed)	70% acetone extract	TPC; 24.2 g PGE/100 g extract TPC; 24.0 g PGE/100 g extract TPC; 15.9 g PGE/100 g extract	DPPH, ORAC	Natural antioxidants for functional foods and nutraceuticals	[102]
*Halopithys incurve* (Red seaweed), *Fucus spiralis, Treptacantha abies-marina* (Brown seaweeds)	Hydroethanolic methanolic and extracts	TPC; 4.8% of DW TPC; 3.1% of DW TPC; 3.9% of DW	DPPH, RAP	NA	[175]
*Ascophyllum nodosum* *Fucus distichus* *Fucus evanescens* (Brown seaweed)	Methanolic extract	TPC; 38.95 PGE% TPC; 30.40 PGE% TPC; 23.85 PGE%	DPPH	NA	[176]
*Sargassum polycystum* (Brown seaweed)	Ethanol/water extract	TPC; 37.41 mg GAE/g DW TFC; 4.54 mg CE/g DW	DPPH, ABTS	NA	[177]
*Ulva intestinalis* (Green seaweed)	Dichloromethane extract	TPC; 197 ± 16 mg GAE/g extract	DPPH, ABTS	Medicine, dietary supplements, cosmetics, and food industries.	[178]
*Acanthophora spicifera* (Red seaweed)	Ethyl acetate extract	TPC; 40.583 GAE; µg mg^−1^ DW	DPPH	Natural antioxidant	[105]
*Himanthalia elongate* (Brown seaweed)	60% methanolic extract	TPC; 286.0 mg GAE/g TFC; 109.8 mg QE/g Condensed tannin; 35.6 mg CE/g	DPPH, FRAP, FCA, inhibition of lipid peroxidation, hydrogen peroxide scavenging activity	Natural food preservative or nutraceutical	[179]
*Himanthalia elongate* (Brown seaweed)	Ethanol/water extract	TPC; 548.33 mg AG/100 g seaweed. Phloroglucinol, Gallic Acid, Catechin, Rutin, Gentisic Acid, Chlorogenic Acid, Caffeic Acid, Coumaric, Ferulic, Myricetin and Quercetin	DPPH	NA	[180]
*Kappaphycus alvarezii* (Red seaweed)	1% Formic acid extracts	TPC; 40 mg (100 g)^−1^ GAE TFC; 60 mg (100 g)^−1^ CE	DPPH, ABTS	NA	[92]
*Laurencia obtuse* (Red seaweed)	Ethanolic extract	TPC; 26.23 mg GAE/g seaweed	ABTS, TAA	NA	[94]
*Macrocystis pyrifera* (Brown seaweed)	Aqueous extract	TPC; 200.5 mg (GAE)/100 g DW Phlorotannin; phloroeckol and a tetrameric phloroglucinol	DPPH, TAA	Medicinal foods or therapeutics	[69]
*Sargassum fusiforme* (Brown seaweed)	Ethyl acetate fractions	TPC; 88.48 mg PGE/100 mg extract. fuhalol-type phlorotannins, phlorethols, fucophlorethols and eckol-type phlorotannins.	DRSA, FRAP	Marine antioxidants	[64]
*Ascophyllum nodosum* (Brown seaweed)	75% (*v/v* aq.) 1,3-propanediol solvent extract	TPC; 100 mg/ (PEG/g) DW	DPPH	NA	[181]

NA—Not available, TPC—Total phenolic content, TFC—Total flavonoid content, TPhC—Total phlorotannin content, GAE—Gallic acid equivalence, CE—Catechin equivalents, PGE—Phloroglucinol equivalents, DW—Dry Weight, ABTS—radical cation decoloration, DPPH—free radical scavenging activity, FRAP—Ferric reducing antioxidant power assay, ORAC—Oxygen radical absorbance capacity, FCA—Ferrous ion chelating ability, CAA—Cellular antioxidant activity, TAC—Total antioxidant capacity, RAP—Reducing activity power, TAA—Total antioxidant activity, TEAC—Trolox equivalent antioxidant capacity, DRSA—DPPH radical-scavenging activity, LMW—Low molecular weight. Search keywords: seaweed phenolics, phlorotannins, antioxidant activity, DPPH, FRAP, TAA, ABTS, food preservative, fish oil stability; years of searching: 2000–2022.

**Table 5:** Cross-linking properties of phenolic compounds and their properties in protein–polyphenol complex (e.g., microencapsulation, hydrogels, emulsions).

Phenolic Compound	Biopolymers	Morphological Characteristics	Physiochemical Characteristics	Application	Reference
Tannic acid	Gelatin-high methyl pectin	Rough and irregular shape, Average particle size (47 µm)	Improved melting and gelling points, thermal stability, encapsulation efficacy (75%)	Peppermint Oil microencapsulation	[209]
Caffeic, Chlorogenic, Ferulic, Rutin, white grape juice, Instant coffee	Gelatin-pectin	NA	Reduced swelling, fewer free amino groups, lipophilicity, thermal stability up to 200 °C	Gelatin-pectin microparticles	[214]
Tannic acid–oxidized and non-oxidized form	Fish gelatin-gum arabic	NA	Improved gelling ability and mechanical properties	Complex coacervate gel	[212]
Tannic acid	Sodium caseinate	NA	Altered secondary structure of SC, high antioxidative properties	Complex coacervate gel	[215]
Caffeic, Tannic acid Oxidized form	Gelatin	NA	Decreased molecular mobility of hydrogels, thermal stability	Gelatin film (Insoluble hydrogels)	[216]
Tannic acid	Gelatin–gum arabic	Spherical in shape, mean cluster size 116.80 μm	High encapsulation efficiency (84%), sustained release of AITC (46% after 2h and 48% in 6h)	Allyl isothiocyanate (AITC) encapsulation	[217]
*Fructus Chebulae* extract (TPC; 360 μg polyphenols/g of gelatin)	Gelatin	Compact surface	Thermal stability, reduced swelling	Gelatin hydrogels	[210]
Tannic acid-oxidized form	Gelatin-flaxseed mucilage	Fine to less firm structure	High encapsulation efficacy (>90% *w/w*) and loading capacity, high stability, controlled release of oil	Flaxseed oil encapsulation	[213]
Ferulic acid, Tannin acid	Gelatin	Smooth surface, increased thickness of layers and interlayer space	High mechanical strength, decrease swelling ratios,	Gelatin film	[208]
Tea polyphenol (Catechin, Epicatechin, Epicatechin gallate, Epigallocatechin gallate)	Milk *β*-lactoglobulin	NA	Structural stabilization via an increase in β-sheet and α-helix	Milk β-lactoglobulin complexes	[218]

NA—Not available; TPC—Total phenolic content. Search keywords: seaweeds, phenolics, phlorotannins, cross-linker, binder, microcaps, encapsulation; hydrogel, biopolymers; Years of searching: 2000–2022.

## Data Availability

Not applicable.
